# The ATP-binding cassette transporter A1 regulates phosphoantigen release and Vγ9Vδ2 T cell activation by dendritic cells

**DOI:** 10.1038/ncomms15663

**Published:** 2017-06-05

**Authors:** Barbara Castella, Joanna Kopecka, Patrizia Sciancalepore, Giorgia Mandili, Myriam Foglietta, Nico Mitro, Donatella Caruso, Francesco Novelli, Chiara Riganti, Massimo Massaia

**Affiliations:** 1 Dipartimento di Biotecnologie Molecolari e Scienze della Salute, Università degli Studi di Torino, Via Nizza 52, Torino 10126, Italy; 2 Centro di Ricerca in Medicina Sperimentale (CeRMS), AOU Città della Salute e della Scienza di Torino, Via Santena 5, Torino 10126, Italy; 3 Dipartimento di Oncologia, Università degli Studi di Torino, Via Santena 5/bis, Torino 10126, Italy; 4 Centro Interdipartimentale di Ricerca per le Biotecnologie Molecolari (CIRBM), Via Nizza 52, Torino 10126, Italy; 5 Dipartimento di Scienze Farmacologiche e Biomolecolari, Università degli Studi di Milano, Via Balzaretti 9, Milano 20133, Italy; 6 SC. Ematologia, AO S. Croce e Carle, Via Michele Coppino 26, Cuneo 12100, Italy

## Abstract

Vγ9Vδ2 T cells are activated by phosphoantigens, such as isopentenyl pyrophosphate (IPP), which is generated in the mevalonate pathway of antigen-presenting cells. IPP is released in the extracellular microenvironment via unknown mechanisms. Here we show that the ATP-binding cassette transporter A1 (ABCA1) mediates extracellular IPP release from dendritic cells (DC) in cooperation with apolipoprotein A-I (apoA-I) and butyrophilin-3A1. IPP concentrations in the supernatants are sufficient to induce Vγ9Vδ2 T cell proliferation after DC mevalonate pathway inhibition with zoledronic acid (ZA). ZA treatment increases ABCA1 and apoA-I expression via IPP-dependent LXRα nuclear translocation and PI3K/Akt/mTOR pathway inhibition. These results close the mechanistic gap in our understanding of extracellular IPP release from DC and provide a framework to fine-tune Vγ9Vδ2 T cell activation via mevalonate and PI3K/Akt/mTOR pathway modulation.

## Abstract (web summary)

γδT cells are activated by phosphoantigens, and ABCA1 is involved in cholesterol transport. Here the authors link these ideas to show that ABCA1, apoA-I and BTN3A1 regulate extracellular phosphoantigen release by dendritic cells, and implicate ABCA1 in mevalonate-mediated activation of Vγ9Vδ2 T cells.

Vγ9Vδ2 T cells are activated by phosphoantigen that are generated in the mevalonate and non-mevalonate pathway of microbial pathogens. The mevalonate pathway of mammalian cells also generates phosphoantigens such as isopentenyl pyrophosphate (IPP), which activate Vγ9Vδ2 T cells almost as efficiently as microbial phosphoantigens^1^,^2^. Vγ9Vδ2 T cells recognize tumour cells that release mevalonate pathway–derived phosphoantigens as IPP^1^. In addition, Vγ9Vδ2 T cells are specifically activated by antigen-presenting cells, such as dendritic cells (DC), particularly when intracellular IPP generation is boosted with zoledronic acid (ZA), which is an inhibitor of the farnesyl pyrophosphate synthase (FPPS) in the mevalonate pathway^3^,^4^,^5^. How IPP is released in the extracellular microenvironment and delivered to Vγ9Vδ2 T cells is unknown.

CD277/butyrophilin-3A1 (BTN3A1) is a type I glycoprotein that has a central function in phosphoantigen-induced Vγ9Vδ2 T cell activation (reviewed in Harly *et al*.)^4^. Two mechanisms have been hypothesized: the ‘allosteric model' proposed by Harly *et al*.^6^ and ‘antigen-presenting model' by Vavassori *et al*.^7^ In the first model, the interaction between intracellular phosphoantigens and the intracellular B30.2 domain of BTN3A1 induces conformational changes of the extracellular domain, which are sensed by Vγ9Vδ2 T cells and cause their activation. In Vavassori's model, intracellular phosphoantigens are exported to the extracellular microenvironment by an unidentified plasma-membrane-associated transporter and presented to Vγ9Vδ2 T cells by the BTN3A1 extracellular domain. Most data currently available are in favour of the allosteric model^8^,^9^, but it is currently unknown whether BTN3A1 is also involved in extracellular phosphoantigen release.

Ecto-F1-ATPase and apolipoprotein A-I (apoA-I) are additional important molecules involved in Vγ9Vδ2 T cell activation^10^,^11^,^12^,^13^. F1-ATPase can bind both apoA-I and triphosphoric acid 1-adenosin-59-yl ester 3-(3-methylbut-3-enyl) ester (ApppI), which is an adenylated derivative of IPP that accumulates in ZA-treated cells. IPP can be released from ApppI by nucleotide pyrophosphatase causing the activation of Vγ9Vδ2 T cells^11^. Soluble apoA-I can improve Vγ9Vδ2 T cell recognition of tumour cells, which overproduce IPP (that is, after ZA treatment), whereas low levels of circulating apoA-I in chronic inflammation are associated with a low activation state of Vγ9Vδ2 T cells^11^,^12^,^13^.

apoA-I is required for the assembly of nascent high-density lipoprotein (HDL) particles, which mediate the reverse cholesterol transport^14^. apoA-I binds the extracellular domain of the ATP-binding cassette transporter A1 (ABCA1). ABCA1 is a member of the ABC transmembrane transporter family, which is abundant in the liver, the gastrointestinal tract and macrophages. ABCA1 effluxes cholesterol and phospholipids to lipid-poor/lipid-free apoA-I. During the HDL assembly process, ABCA1 cooperates with the ATP-binding cassette transporter G1 (ABCG1) and the scavenger receptor-BI (SR-BI), which are two transporters that are involved in the delivery of cholesterol and choline-rich phospholipids to HDL^14^. Interestingly, ABCA1 also effluxes α-tocopherol^15^, which is a molecule that contains multiple isoprenoid units; however, it has never been reported whether ABCA1 or any other ABC transporter are involved in the extracellular release of IPP or other isoprenoids.

On the basis of these observations, here we test the hypothesis that ABCA1, apoA-I, and BTN3A1 are involved in extracellular IPP release. We demonstrate that ABCA1, in cooperation with apoA-I and BTN3A1, has a major function in extracellular IPP release from ZA-treated DC. Supernatants from ZA-treated DC contain sufficient IPP to induce Vγ9Vδ2 T cell proliferation even after removal of ZA-treated DC. These data show that ABCA1 has a function that may alter our understanding of the relationship between lipid metabolism and immune function.

## Results

### Soluble IPP-induced Vγ9Vδ2 T cell proliferation

**Figure 1:**
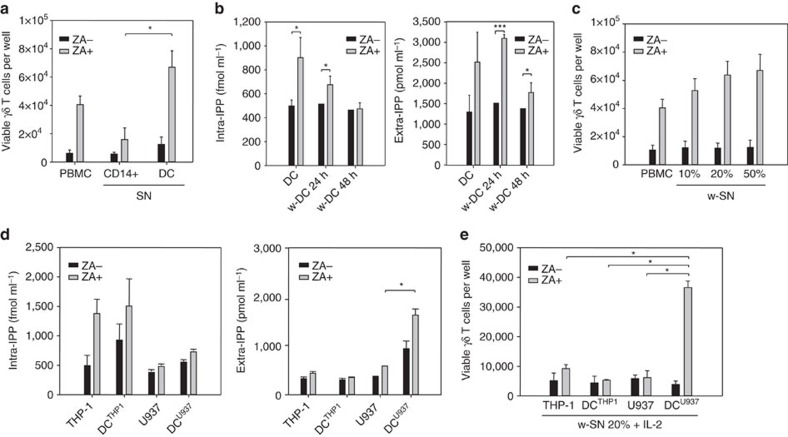
Vγ9Vδ2 T cell proliferation is induced by extracellular IPP. (**a**) Vγ9Vδ2 T cell proliferation after 7-day PBMC stimulation with ZA or supernatant (SN) retrieved from untreated (ZA-) and ZA-treated (ZA+) CD14+ cells and DC. Supernatant from ZA-treated DC induced significantly higher Vγ9Vδ2 T cell proliferation than PBMC stimulation with supernatant from ZA-treated CD14+ cells (*P<0.05; Wilcoxon–Mann–Whitney). The bars represent the mean value±s.e.m. of eight experiments. (**b**) Intracellular IPP concentrations in untreated (ZA-) and ZA-treated (ZA+) washed DC (w-DC) and extracellular IPP concentrations in corresponding SN after washing and incubation for 24 and 48 h without further ZA stimulation. ZA-treated w-DC released significant IPP amounts for 24 h after ZA removal. The bars represent the mean value±s.e.m. of ten experiments (*P<0.05; *P<0.001; ANOVA). (**c**) Vγ9Vδ2 T cell proliferation after 7-day PBMC stimulation with ZA or with serial dilutions of supernatants (w-SN) retrieved from untreated (ZA-) and ZA-treated (ZA+) w-DC. The bars represent the mean value±s.e.m. of eight experiments. (**d**) Intracellular and extracellular IPP levels generated in untreated (ZA-) and ZA-treated (ZA+) THP-1 cells, DC^THP1^, U937 cells and DC^U937^. ZA-treated THP-1 cells and DC^THP1^ generated high amounts of intracellular IPP, which was not released in the supernatant. Conversely, ZA-treated DC^U937^ released extracellular IPP amounts that were close to the extracellular IPP amounts released by ZA-treated DC. Bars represent the mean value±s.e.m. of eight experiments (*P<0.05; ANOVA). (**e**) Vγ9Vδ2 T cell proliferation after 7-day PBMC stimulation with supernatant obtained from untreated (ZA-) and ZA-treated (ZA+) THP-1 cells, DC^THP1^, U937 cells and DC^U937^. Only SN from ZA-treated DC^U937^ induced Vγ9Vδ2 T cell proliferation. The bars represent the mean value±s.e.m. of five experiments (*P<0.05; Wilcoxon–Mann–Whitney). ANOVA, analysis of variance.

Figure 1a shows the proliferation of Vγ9Vδ2 T cells after 7-day stimulation of peripheral blood mononuclear cells (PBMC) from healthy donors with supernatants obtained from autologous monocytes (CD14+) or DC that are left untreated or treated with ZA for 24 h. As previously reported, supernatants from ZA-treated DC induced significant Vγ9Vδ2 T cell proliferation^3^.

In a second set of experiments, DC were incubated for 24 h with or without ZA and washed to clear supernatants from any residual ZA. Washed DC were re-plated for an additional 24 and 48 h in the absence of any other treatment, and intracellular and extracellular IPP levels were evaluated as previously reported (Fig. 1b)^3^. ZA-treated washed DC released significant amounts of IPP for the first 24 h and their supernatants induced a dose-dependent proliferation of Vγ9Vδ2 T cells (Fig. 1c).

The same set of experiments was performed using unmanipulated or DC-like cells that were generated from the acute monocytic leukaemia THP-1 cell line (DC^THP1^) and the histiocytic lymphoma U937 cell line (DC^U937^)^16^,^17^,^18^. DC^THP1^ and DC^U937^ have been shown to perform in many functional assays as well as the conventional counterparts generated from CD14+ cells. ZA-treated THP-1 cells and DC^THP1^ produced significant amounts of intracellular IPP, such as ZA-treated DC (Fig. 1b), whereas U937 cells and DC^U937^ produced lower amounts, which were unaffected by ZA treatment (Fig. 1d). Extracellular IPP release showed an opposite pattern: THP-1 cells and DC^THP1^ did not release extracellular IPP even after ZA treatment, whereas ZA-treated DC^U937^ released intermediate levels (Fig. 1d) that were similar to the levels released by ZA-treated DC (Fig. 1b). The different IPP concentrations in the supernatants translated into a different ability to induce Vγ9Vδ2 T cell proliferation (Fig. 1e).

### Increased ABCA1 and apoA-I expression after ZA treatment

**Figure 2:**
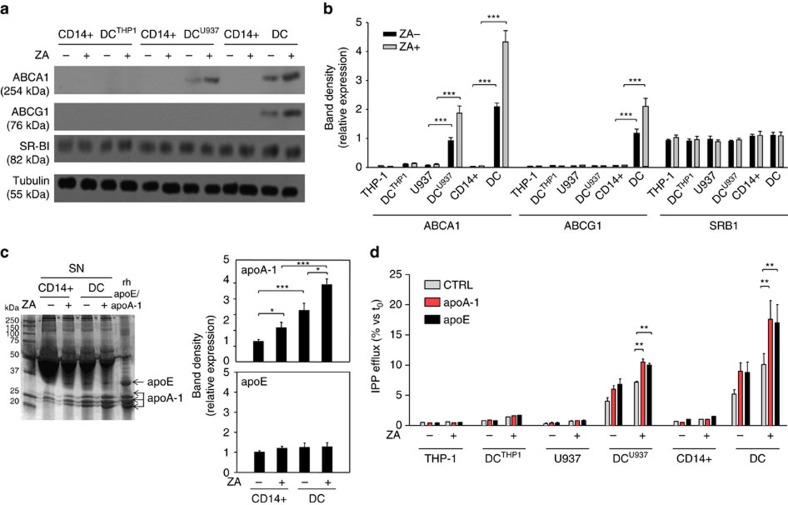
ZA treatment increases ABCA1 and apoA-1 expression. (**a**) Western blot analysis of ABCA1, ABCG1 and SR-BI expression in untreated (ZA-) and ZA-treated (ZA+) THP-1 cells, U937 cells, CD14+ cells and corresponding DC subsets (DC^THP1^, DC^U937^, DC). ABCA1 was already detectable in DC^U937^ and DC and its expression was upregulated by ZA. β-tubulin was employed to check the equal protein loading per lane. The results are obtained from one representative experiment of three experiments. (**b**) Pooled data obtained by densitometric analysis with ImageJ software (http://imagej.nih.gov/ij/). The results are expressed as arbitrary units (THP-1 cells and DC^THP1^, *n*=3; U937 cells and DC^U937^, *n*=3; CD14+ cells and DC, *n*=14) (***P<0.001; ANOVA). (**c**) Supernatants from untreated (ZA-) or ZA-treated (ZA+) CD14+ cells and DC were resolved by SDS–PAGE and stained with Coomassie brilliant blue. A concentration of 1 μg ml^−1^ recombinant human apoE or apoA-I were employed as standards. Densitometric analyses of bands identified as apoE or apoA-I by MALDI-TOF/MS were performed with ImageJ software. The results are expressed as arbitrary units. The gel is representative of one out of the three experiments (*P<0.05, ***P<0.001; ANOVA). (**d**) Extracellular IPP released in the supernatants retrieved from untreated (ZA-) or ZA-treated (ZA+) CD14+ cells, U937 cells, THP-1 cells and corresponding DC subsets (DC, DC^U937^, DC^THP1^) in the absence (CTRL) or presence of human recombinant 1 μg ml^−1^ apoA-I or apoE added during the last 24 h of incubation. apoA-I and apoE increased the IPP release in DC and DC^U937^ but did not increase the IPP release in DC^THP1^. The bars represent the mean value±s.e.m. of three experiments (**P<0.01; ANOVA). ANOVA, analysis of variance. MALDI-TOF/MS, matrix-assisted laser desorption ionization-time-of-flight/mass spectrometry. SDS-PAGE: sodium dodecyl sulfate-polyacrilammide gel electophoresis.

ABCA1, ABCG1 and SR-BI expression were investigated under baseline conditions and after ZA treatment (Fig. 2a). THP-1, U937 and CD14+ cells exhibited no detectable ABCA1 or ABCG1 levels, irrespective of ZA treatment. ZA-treated DC^THP1^ also exhibited no expression, whereas untreated DC^U937^ and DC already exhibited detectable ABCA1 expression, which increased after ZA treatment. DC were the only cells to show detectable ABCG1 expression, which was upregulated by ZA treatment. SR-BI expression was uniformly detectable in all cell preparations and unaffected by ZA treatment. Pooled data with statistical analyses are shown in Fig. 2b.

apoA-I binds to the extracellular domain of ABCA1 to load cholesterol and phospholipids that are released in the reverse cholesterol transport. apoE is also involved in the same process but it binds to ABCG1. We performed a matrix-assisted laser desorption ionization-time-of-flight/mass spectrometry (MALDI-TOF/MS) analysis to investigate whether apoA-I and apoE were present in the supernatant of CD14+ cells and DC (Supplementary Table 1). Two bands of 36.1 and 30.7 kD, which correspond to apoE and apoA-I, were identified (Fig. 2c, left; Supplementary Fig. 1; Supplementary Table 1). ZA treatment did not modify apoE levels, whereas it increased apoA-I expression (Fig. 2c, right). These data indicate that ZA-induced mevalonate pathway inhibition increases the extracellular release of both IPP and apoA-I in both CD14+ and DC.

To further explore the relationship among IPP, apoA-I, and apoE, extracellular IPP was quantified in the supernatant of untreated and ZA-treated THP-1 cells, U937 cells, CD14+ cells, DC^THP1^, DC^U937^, and DC after incubation with recombinant human apoA-I and apoE and pulsing with [^14^C]-IPP. As shown in Fig. 2d, exogenously added apoA-I and apoE promoted extracellular IPP release only in cells with detectable ABCA1 expression, that is, untreated and ZA-treated DC^U937^ and DC.

To further build on the relationship between IPP and ABCA1, we correlated intracellular and extracellular IPP levels with ABCA1, ABCG1 and SR-B1 expression in a series of haematologically derived neoplastic and non-neoplastic cells (Supplementary Fig. 2A, Supplementary Fig. 2B). A strong correlation was detected between ABCA1 levels and extracellular IPP levels (*r*^2^=0.98), whereas no correlation between intracellular IPP levels and ABCA1 levels was identified (Supplementary Fig. 2C). ZA treatment upregulated ABCA1 expression only in cells with detectable ABCA1 under baseline conditions, such as the myeloma cell line SKMM1, primary multiple myeloma cells (CD138+ MM) and bone marrow stromal cells (BMSC) derived from multiple myeloma patients (Supplementary Fig. 3).

**Figure 3:**
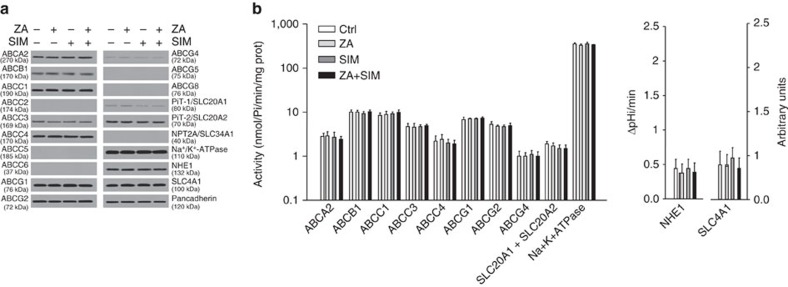
ZA treatment does not affect other transporters in ZA and SIM-treated DC. Levels of the indicated plasma-membrane-associated proteins are evaluated by western blotting in biotinylated extracts from untreated or 1 μM ZA and/or simvastatin (SIM) treated DC. Pancadherin is employed as a control of equal protein loading. The blots are representative of one out of the three experiments. (**b**) Activities of the indicated plasma-membrane-associated proteins are measured after immunopurification from the biotinylated extracts of DC treated, as shown in **a**. The results are expressed as nmoles hydrolysed phosphate (Pi)/min/mg proteins according to the previously prepared titration curve. The NHE1 activities and SLC4A1 activities are fluorimetrically measured, and the results are expressed as ΔpHi/min and arbitrary units, respectively. The bars represent the mean±s.e.m. of three experiments. None of the differences is statistically significant.

To eliminate the possibility that ZA-induced ABCA1 upregulation was not merely due to modifications of cholesterol content in the plasma-membrane and associated with non-specific changes in other membrane proteins, we compared the expression levels and activities of unrelated transporters and channels in untreated and DC treated with ZA and/or simvastatin. Simvastatin is a selective inhibitor of 3-β-hydroxy-β-methylglutaryl coenzyme A reductase (HMGR) upstream to FPPS in the mevalonate pathway that strongly reduces cholesterol synthesis^3^. The results in Fig. 3 indicate that neither the expression nor the activity of other ABC transporters that are involved in cholesterol and lipid metabolites efflux (ABCA2, ABCB1, ABCC1, ABCC2, ABCC3, ABCC4, ABCC5, ABCC6, ABCG2, ABCG4, ABCG5, ABCG8), phosphate transporters potentially involved in pyrophosphate transport (PiT-1/SLC20A1, PiT-2/SLC20A2, NPT2A/SLC34A1), and other ubiquitous and unrelated pumps or channels (Na^+^/K^+^-ATPase, Na^+^/H^+^ exchanger 1/NHE1, Cl^−^/HCO_3_^−^ transporter/SLC4A1) were modified by ZA or simvastatin treatment.

### IPP is released by ABCA1 in cooperation with BTN3A1

**Figure 4:**
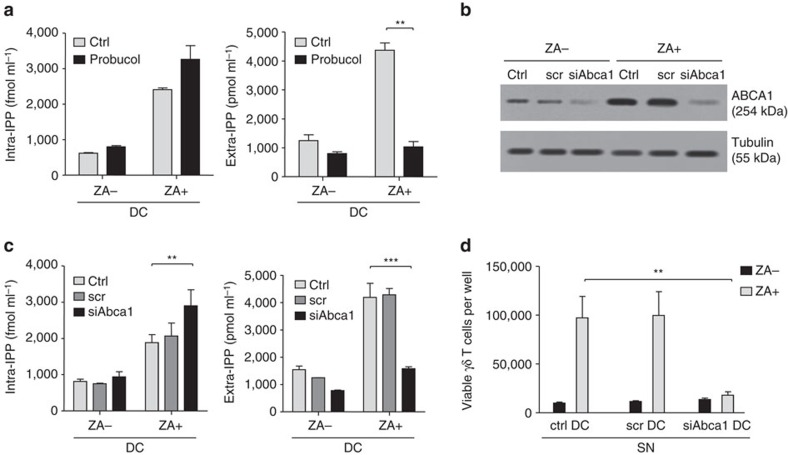
ABCA1 has a pivotal function in extracellular IPP release. (**a**) Intracellular and extracellular IPP levels generated by untreated (ZA-) and ZA-treated (ZA+) DC incubated for 24 h in fresh medium (CTRL) or with 10 μM probucol to inhibit ABCA1 function. Probucol marginally increased intracellular IPP but significantly decreased extracellular IPP release in ZA-treated DC (**P<0.01; ANOVA). The bars represent the mean±s.e.m. of four experiments. (**b**) Western blot analysis of ABCA1 expression in untreated (ZA-) and ZA-treated (ZA+) DC after *Abca1-*silencing with siRNA (scrambled [scr] non-targeting siRNA; siAbca1: *Abca1*-silencing siRNA). The blots are representative of one out of the three experiments. β-tubulin was employed as the control of equal protein loading. (**c**) Intracellular and extracellular IPP levels in DC incubated for 48 h in fresh medium (CTRL) or in the presence of scr or *Abca1*-silencing siRNA (siAbca1). ZA (1 μM) was added in the last 24 h. Extracellular IPP release is significantly lower in *Abca1*-silenced ZA-treated DC. Bars represent the mean±s.e.m. of six experiments (**P<0.01; ***P<0.001; ANOVA). (**d**) Vγ9Vδ2 T cell proliferation after 7-day PBMC stimulation with supernatants from DC treated as reported in **c**. Supernatant from *Abca1*-silenced ZA-treated DC is unable to induce Vγ9Vδ2 T cell proliferation (**P<0.01; Wilcoxon–Mann–Whitney). The bars represent the mean±s.e.m. of six experiments. ANOVA, analysis of variance.

Probucol is a functional ABCA1 inhibitor^19^. Probucol incubation of untreated and ZA-treated DC for 24 h marginally increased intracellular IPP levels and completely abrogated ZA-induced extracellular IPP release (Fig. 4a).

To confirm the role of ABCA1, siRNA were employed to silence *Abca1* in untreated and ZA-treated DC. The efficacy and specificity are shown in Fig. 4b. As expected, *Abca1*-silencing marginally increased intracellular IPP generation, whereas it completely abrogated ZA-induced extracellular IPP release (Fig. 4c). The inability to induce Vγ9Vδ2 T cell proliferation provided the functional demonstration that supernatants from ZA-treated *Abca1*-silenced DC were devoid of IPP (Fig. 4d). These results indicate that ABCA1 has a major role in the extracellular IPP release from ZA-treated DC.

**Figure 5:**
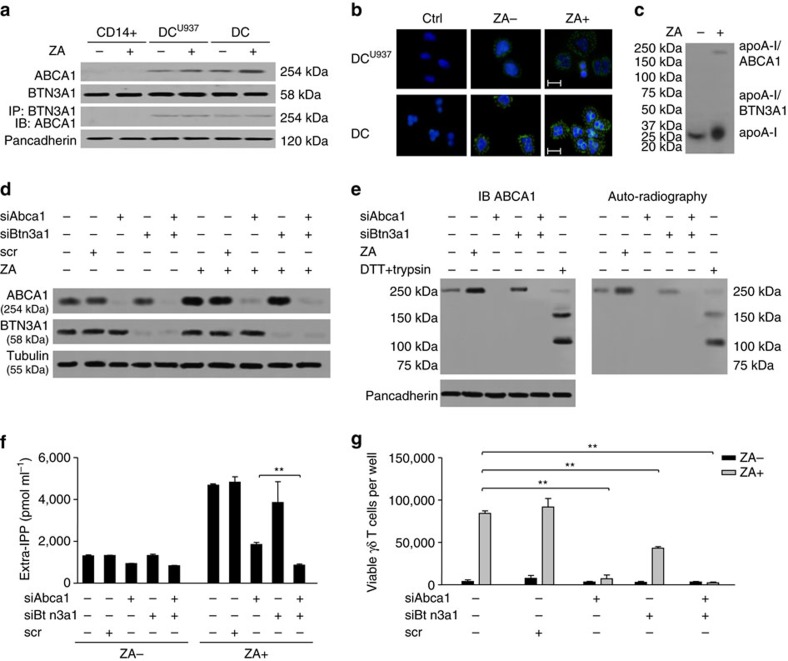
ABCA1 interactions with BTN3A1, apoA-I and IPP. (**a**) ABCA1 and BTN3A1 co-immunoprecipitate in untreated and ZA-treated DC^U937^ and DC but not in CD14+ cells, and ZA treatment does not modify BTN3A1 expression. Pancadherin is employed as a control of equal protein loading (one out of three blots). (**b**) PLA of ABCA1-BTN3A1 interaction by confocal laser-scanning microscopy (ocular lens: × 10; objective: × 63). Ctrl: cells without primary antibodies. Scale bar, 10 μm; blue: nuclear staining (DAPI); green: ABCA1/BTN3A1 interaction (one out of the three experiments). (**c**) apoA-I is physically associated with ABCA1, not with BTN3A1. The expected molecular weight of apoA-I, ABCA1/apoA-I and BTN3A1/apoA-I are shown (one out of the three experiments). (**d**) ABCA1 and BTN3A1 expression in DC after incubation with siRNA for *Abca1* (siABca1), *Btn3a1* (siBtn3a1) or with scrambled non-targeting siRNA (scr). β-tubulin was employed as control of equal protein loading (*n*=3). (**e**) IPP is physically associated with ABCA1 in untreated and ZA-treated DC. The two bands of 150 and 100 kDa in the dithiothreitol (DTT)- and trypsin-treated DC lane are detected with an antibody specific for the N-terminal extracellular loop of ABCA1 in immunoblotting (IB; left). Autoradiography signal of the IPP-ABCA1 interaction (right). Pancadherin is employed as a control of equal protein loading (one out of three experiments). (**f**) Extracellular IPP release in *Abca1-* and/or *Btn3a*1-silenced DC left untreated (ZA-) or after ZA treatment (ZA+). The bars represent the mean±s.e.m. of three experiments (**P<0.001, Wilcoxon–Mann–Whitney). (**g**) Vγ9Vδ2 T cell proliferation after 7-day PBMC stimulation with supernatants from *Abca1-* and/or *Btn3a1-*silenced DC. The bars represent the mean±s.e.m. of three experiments (**P<0.01; Wilcoxon–Mann–Whitney).

BTN3A1 has been reported to be involved in the phosphoantigen-induced activation and recognition of target cells by Vγ9Vδ2 T cells^5^,^6^. To explore any relationship between ABCA1 and BTN3A1, BTN3A1 expression was investigated by flow cytometry in CD14+ cells, DC^THP1^, DC^U937^, and DC without detecting any difference under baseline conditions or after ZA treatment (Supplementary Fig. 2D). BTN3A1 and ABCA1 were co-immunoprecipitated in DC^U937^ and DC, which were the only cells to express detectable amounts of ABCA1 (Fig. 5a). ZA treatment increased ABCA1 expression but did not modify BTN3A1 expression. The physical interaction between ABCA1 and BTN3A1 was confirmed in DC^U937^ and DC by the proximity ligation assay (PLA) (Fig. 5b). Next, we tested the presence of interactions between apoA-I and ABCA1 or BTN3A1. After crosslinking of apoA-I to cell surface proteins and immunoprecipitation with anti-apoA-I antibody, we found that apoA-I and ABCA1 are physically associated as previously reported^20^, whereas no interaction was detected between apoA-I and BTN3A1 (Fig. 5c).

To determine the reciprocal contributions to extracellular IPP release, BTN3A1 and ABCA1 were alternatively or concurrently silenced in untreated and ZA-treated DC. The efficacy and specificity of siRNA are shown in Fig. 5d: untreated DC (ZA−) expressed detectable levels of ABCA1, which further increased in ZA-treated DC (ZA+) and disappeared in untreated and ZA-treated *Abca1*-silenced and *Abca1*+*Btn3a1*-silenced DC, whereas ABCA1 expression was not suppressed in *Btn3a1*-silenced DC. To determine whether IPP was physically associated with ABCA1, DC were treated as shown in Fig. 5d, radiolabelled with 1 μM [^14^C]-IPP, and chemically crosslinked after extensive washing. ABCA1 was immunoprecipitated by plasma-membrane extracts, resolved by sodium dodecyl sulfate–polyacrylamide gel electrophoresis (SDS–PAGE) and probed with an anti-ABCA1 antibody that recognizes the N-terminal extracellular loop (Fig. 5e, left) or subjected to autoradiography (Fig. 5e, right). As internal control, biotinylated plasma-membrane extracts from ZA-treated DC were subjected to limited digestion to obtain distinct extracellular and intracellular ABCA1 fragments^21^,^22^. Autoradiography showed a positive signal in untreated DC coincidental with the expected ABCA1 molecular weight, which was increased in ZA-treated DC. The radioactive signal was still present in *Btn3a1*-silenced DC, whereas it was not detectable in *Abca1*-silenced and *Abca1/Btn3a1*-silenced DC. In dithiothreitol+trypsin-treated DC, the radioactive signal was coincidental with the 100 kDa band and, to a lesser extent, with the 150 kDa band, which suggests that IPP is physically associated with the N-terminal extracellular domain of ABCA1.

The functional consequences of *Abca1*-silencing and *Btn3a1*-silencing are shown in Fig. 5f,g. Minimal variations of extracellular IPP levels were induced when *Abca1* and/or *Btn3a1* were silenced in the absence of ZA-treatment (Fig. 5f). Conversely, ZA-treated *Abca1*-silenced DC showed a significant reduction in the ability to release extracellular IPP, whereas ZA-treated *Btn3A1-*silenced DC were only marginally affected. When both genes were silenced, further reduction in extracellular IPP release was observed (Fig. 5f). As expected, Vγ9Vδ2 T cell proliferation induced by supernatants from *Abca1*- and/or *Btn3A1-*silenced ZA-treated DC matched the extracellular IPP concentrations (Fig. 5g). These data indicate that ABCA1 plays a major role in extracellular IPP release; this function is facilitated by physical interactions with IPP, apoA-I and BTN3A1.

### ZA increases IPP efflux via LXRα-mediated Abca1 activation

**Figure 6:**
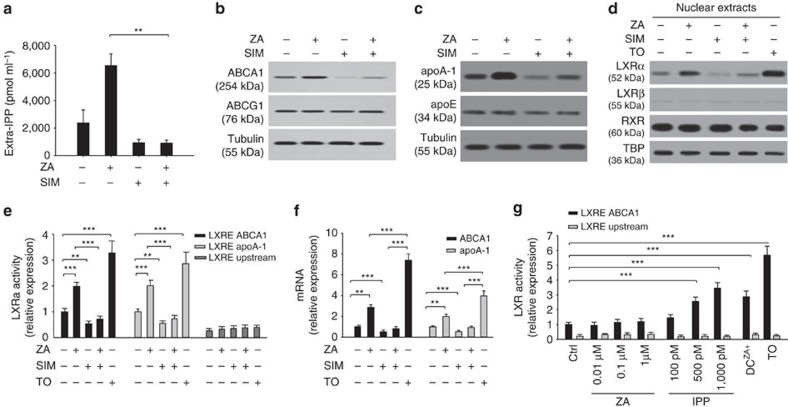
Regulation of ABCA1 and apoA-I expression via LXRα nuclear translocation. (**a**) Extracellular IPP release in DC left untreated or incubated with 1 μM ZA and/or simvastatin (SIM, 1 μM). Simvastatin abrogated ZA-induced extracellular IPP release. Bars represent the mean±s.e.m. of four experiments (**P<0.01; ANOVA). (**b**) ABCA1 expression in experimental conditions as in **a**. ZA increased ABCA1 expression, whereas simvastatin showed the opposite effect and abrogated ZA-induced ABCA1 upregulation. (**c**) apoA-I expression in experimental conditions, as shown in **a**. ZA increased apoA-1 expression, whereas simvastatin exhibited the opposite effect and abrogated ZA-induced apoA-I upregulation. apoE was unaffected by ZA and/or simvastatin treatments. (**d**) LXRα, LXRβ and RXR levels were measured in nuclear extracts from DC incubated in the absence (−) or presence (+) of ZA and/or simvastatin. The LXRα activator TO-901317 (TO, 100 nM for 24 h) was employed as a positive control. ZA increased LXRα protein levels and this effect was antagonized by SIM. LXRβ and RXR were unaffected by ZA and/or simvastatin treatment. β-tubulin and TBP are employed as controls of equal protein loading as indicated. The blots are representative of one out of thee experiments (**b**–**d**). (**e**) Activity of *Abca1* and *apoA-I* promoters in the experimental conditions, as shown in **d**. ZA increased, whereas simvastatin decreased and antagonized the ZA-induced LXRα transcriptional activity of *Abca1* and *apoA-I* promoters. The bars represent the mean±s.e.m. of three experiments (**P<0.01; ***P<0.001; ANOVA). (**f**) *Abca1* and *apoA-I* mRNA levels in the experimental conditions, as shown in **d**. ZA increased the *Abca1* and *apoA-I* mRNA levels, whereas simvastatin had the opposite effect and antagonized ZA-induced upregulation. The bars represent the mean±s.e.m. of three experiments (**P<0.01; ***P<0.001; ANOVA). (**g**) Evaluation of LXRα binding to LRE in the *Abca1* promoter. ZA did not modify LXRα transcriptional activity, whereas IPP induced a dose-dependent upregulation of LXRα-dependent *Abca1* transcription. LXRα transcriptional activity in ZA-treated DC and TO-induced LXRα transcriptional activity are reported as positive internal controls. The bars represent the mean±s.e.m. of four experiments (*P<0.001; ANOVA). ANOVA, analysis of variance.

Simvastatin inhibits the mevalonate pathway upstream to FPPS^3^. As expected, simvastatin decreased extracellular IPP release in both untreated and ZA-treated DC (Fig. 6a). Interestingly, simvastatin also reduced ABCA1 (Fig. 6b) and apoA-I expression (Fig. 6c). These data confirm the close relationship between the mevalonate pathway, ABCA1, apoA-I and extracellular IPP release.

ABCA1 and apoA-I expressions are controlled by the liver X receptor (LXR)/retinoic acid X receptor (RXR) transcription factor receptor family^23^,^24^. We investigated whether ZA and simvastatin regulated ABCA1 and apoA-I expression via these transcription factors. Untreated DC expressed both LXRα/β and RXR in nuclear extracts (Fig. 6d). ZA treatment increased LXRα protein levels, whereas LXRβ and RXR intracellular distribution was unaffected (Fig. 6d). LXRα bound to *Abca1* and *apoA-I* promoters was increased (Fig. 6e), leading to increased *Abca1* and *apoA-I* mRNA levels (Fig. 6f). These ZA-induced effects were neutralized by simvastatin (Fig. 6d–f). These data indicate that ZA-induced LXRα activity is crucial to increase ABCA1 and apoA-I expression in DC and promotes extracellular IPP release.

To reinforce the role of LXRα, the experiments mentioned above were repeated using THP-1 cells, which are unable to release extracellular IPP, even after DC differentiation and ZA treatment. Unlike DC, LXRα and LXRβ failed to translocate into the nucleus after ZA treatment in THP-1 cells and DC^THP1^ (Supplementary Fig. 4A). As expected, *Abca1* and *apoA-I* mRNA levels remained unchanged by ZA treatment (Supplementary Fig. 4B). These data confirm that LXRα is critical to induce *Abca1* and *apoA-I* transcription and protein expression after ZA-induced IPP accumulation.

To determine whether ZA or IPP was responsible for LXRα activation, genomic DNA was extracted from DC and challenged with increasing ZA and IPP concentrations in the presence of the human recombinant LXRα transcription protein. Only IPP was able to induce a dose-dependent LXRα binding to the LXR responsive element (LRE) in the *Abca1* promoter (Fig. 6g). Optimal LXRα activation was induced by 500 pM IPP, which falls in the range of concentrations detected in the supernatants of ZA-treated DC (Fig. 6g). We tested whether other isoprenoids generated in the mevalonate pathway, such as FPP and GGPP, can also induce LXRα activation and we found that the former had no effect, whereas the latter downregulated LXRα activation (Supplementary Fig. 5) as previously reported^25^.

Next, we investigated whether ZA and/or IPP also increased the enzyme activity of ABCA1 in addition to its expression. Identical aliquots of ABCA1 were immunopurified from the plasma membrane of DC and incubated with increasing concentrations of ZA (Supplementary Fig. 6A) or IPP (Supplementary Fig. 6B). The experimental conditions were established to enable any change in ATPase activity to be referred to differences in enzyme activity instead of protein expression. The results indicate that neither ZA nor IPP were able to directly upregulate ABCA1 ATPase-activity (Supplementary Fig. 6A,B). Next, we compared the ATPase activity of ABCA1 immunopurified from the plasma membrane of untreated and ZA-treated CD14+ cells and DC. Untreated CD14+ cells exhibited the lowest ATPase activity, which remained unaffected by ZA treatment, whereas untreated DC revealed intermediate values, which almost doubled in ZA-treated DC (Supplementary Fig. 6C). The results suggest that the increased ATPase activity in ZA-treated DC is more likely due to increased ABCA1 protein levels than to the direct effect exerted by ZA or IPP on its enzymatic activity.

### PI3K/AkT/mTOR pathway regulates IPP release via LXRα/Abca1

**Figure 7:**
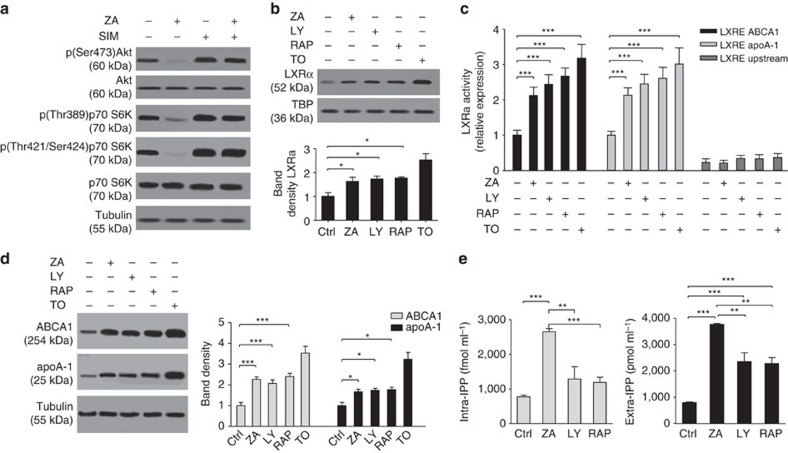
The PI3K/AkT/mTOR pathway regulates IPP release via LXRα/Abca1 activation. (**a**) Targeting the Mev pathway fine-tunes the PI3K/Akt/mTOR signalling pathway. DC were left untreated or incubated with 1 μM ZA or simvastatin (SIM). ZA decreased, whereas simvastatin increased the signalling activity of the PI3K/Akt/mTOR pathway. (**b**) DC were grown for 24 h in the absence (−) or presence (+) of ZA (1 μM), the PI3K inhibitor LY294002 (LY, 200 μM for 24 h ), the mTOR inhibitor rapamycin (RAP, 20 nM for 24 h). The LXRα activator TO-901317 (TO, 100 nM for 24 h) was included as positive control. PI3K/mTOR inhibitors increased LXRα nuclear translocation, such as ZA. Pooled data are obtained by densitometric analysis with ImageJ software (http://imagej.nih.gov/ij/). The results are expressed as arbitrary units (*n*=3) (*P<0.05; ANOVA). (**c**) Evaluation of LXRα binding to LRE sequences in the *Abca1* and *apoA-1* promoters by ChIP assay. ZA, LY294002 and rapamycin increased *Abca1* and *apoA-1* promoters activity. The bars represent the mean±s.e.m. of three experiments (***P<0.001; ANOVA). (**d**) Western blot analysis of ABCA1 and apoA-1 expression in experimental conditions, as shown in **b**. PI3K and mTOR inhibition mimicked ZA treatment and upregulated ABCA1 and apoA-1 expression. β-tubulin and TBP were employed as a control of equal protein loading, as indicated. All blots are representative of out the of three experiments. Pooled data are obtained by densitometric analysis with ImageJ software (http://imagej.nih.gov/ij/). The results are expressed as arbitrary units (*n*=3). (*P<0.05, ***P<0.001; ANOVA). (**e**) Intracellular and extracellular IPP levels in DC after incubation in experimental conditions, as shown in **b**. As expected, PI3K/Akt/mTOR inhibition only increased extracellular IPP. The bars represent the mean±s.e.m. of three experiments (**P<0.01, ***P<0.001; ANOVA). ANOVA, analysis of variance.

Akt suppresses cholesterol efflux by the activation of the mammalian target of rapamycin (mTOR) complex 1 TORC1, whereas the inhibition of mTORC1 by rapamycin promotes cholesterol efflux to apoA-I in an ABCA1-dependent manner^26^. The phosphoinositide 3-kinase (PI3K)/Akt/mTOR signalling pathway is also regulated by the Ras prenylation state, which is dependent on the mevalonate pathway in cancer cells and activated T cells^27^,^28^. To investigate the involvement of the PI3K/Akt/mTOR signalling pathway in ABCA1/apoA-I-mediated extracellular IPP release, DC were incubated with specific PI3K (LY294002) and mTOR (rapamycin) inhibitors (refer to Methods and Supplementary Fig. 7 for technical details). Untreated DC showed detectable phosphorylation activity of both Akt and mTOR, which was very effectively blocked by ZA treatment. Akt and mTOR phosphorylation activities were fully recovered by simvastatin treatment in ZA-treated DC (Fig. 7a), although simvastatin also induces intracellular isoprenoid deprivation and Ras deprenylation^29^,^30^. A possible explanation is that isoprenoid deprivation recovers the activity of the PI3K/Akt/mTOR pathway via induction of the endoplasmic reticulum (ER) stress and unfolded protein responses (UPR), as previously reported^31^. In line with this hypothesis, we have demonstrated that several ER-stress-related genes are significantly upregulated in ZA+simvastatin-treated compared with untreated DC (Supplementary Data 1), as previously reported with ZA or simvastatin in other cell types^32^,^33^.

PI3K and mTOR inhibitors mimicked the effect of ZA by significantly increasing nuclear LXRα protein levels (Fig. 7b), LXRα transcriptional activity (Fig. 7c), and ABCA1 and apoA-I protein expression (Fig. 7d). Unlike ZA, LY294002 and rapamycin did not increase intracellular IPP levels as these inhibitors do not target the mevalonate pathway (Fig. 7e, left). However, both inhibitors increased extracellular IPP release as they upregulated ABCA1 and apoA-I protein expression. However, LY294002 and rapamycin did not achieve ZA-induced extracellular IPP values as they did not induce intracellular IPP accumulation (Fig. 7e, right).

## Discussion

In this study, we confirm that soluble IPP can induce Vγ9Vδ2 T cell activation^3^,^34^ and demonstrate that the plasma-membrane-associated transporter ABCA1 plays a major role in the extracellular IPP release from ZA-treated DC and other cells. apoA-1 and BTN3A1 are also important contributors to this process, which ultimately causes the activation of Vγ9Vδ2 T cells. Although other metabolites downstream to IPP can also activate Vγ9Vδ2 T cells (that is, DMAPP, GPP, FPP and GGPP)^35^, none of these molecules achieved mitogenic concentrations in untreated DC and their levels further decreased after ZA treatment. These data exclude a contribution of these molecules in ZA-induced Vγ9Vδ2 T cell activation.

Cells that are unable to release extracellular IPP in baseline conditions or after ZA-induced intracellular IPP accumulation do not express ABCA1; contrariwise, cells with a high capacity to release intracellular IPP express ABCA1 and further up-regulate its expression after ZA treatment. IPP is physically associated with the N-terminal extracellular domain of ABCA1 and this association is strengthened in ZA-treated cells that have increased intracellular ABCA1 and IPP levels.

ABCA1 upregulation by ZA treatment is associated with an increased apoA-1 concentration in the supernatants. The mutual interaction between ABCA1 and apoA-1 was confirmed by the ability of exogenously added apoA-I to facilitate extracellular IPP release, but only in cells that already express ABCA1 in baseline conditions or after ZA treatment. We cannot exclude *a priori* that other transporters are involved in IPP efflux. However, the unique relationship between ABCA1 upregulation and intracellular IPP accumulation was corroborated by the observation that ZA and simvastatin treatment, which increase IPP and decrease IPP respectively, do not modify the expression of other ABC transporters, phosphate transporters or other ubiquitous and unrelated pumps or channels. These results also eliminated the possibility that ABCA1 expression is upregulated merely because the cholesterol content of plasma membranes or the intracellular concentration of other mevalonate pathway-related metabolites are modified by ZA or simvastatin.

The experiments in which ABCA1 was functionally inhibited with probucol, or protein expression specifically silenced with siRNA, provided direct evidence that ABCA1 plays a major role in extracellular IPP release. Both treatments only slightly increased intracellular IPP concentrations in untreated and ZA-treated DC, probably because downstream enzymes in the mevalonate pathway like IPP isomerase and prenyl transferases intervene to handle IPP accumulation in the absence of ABCA1 upregulation. More importantly, probucol and *Abca1*-silencing abrogated extracellular IPP release. IPP deprivation in the supernatants from ABCA1-inhibited DC was functionally confirmed by the inability to induce Vγ9Vδ2 T cell proliferation.

BTN3A1 is also involved in extracellular IPP release. BTN3A1 is an immunoglobulin superfamily protein playing a key role in Vγ9Vδ2 T cell activation and target recognition by Vγ9Vδ2 T cells^4^,^6^,^7^,^36^,^37^,^38^,^39^. The allosteric/sensor model anticipates that intracellular interactions between the phosphoantigens domain and the B30.2 domain of BTN3A1 induce conformational changes of the transmembrane domain that are sensed by Vγ9Vδ2 T cells, which cause their activation. The presentation model predicts that BTN3A1 functions as an antigen-presenting molecule for Vγ9Vδ2 T cells by extracellularly binding phosphoantigens^7^. The two models are not mutually exclusive and both models contemplate the possible contribution of a second protein that has yet to be characterized^4^,^40^. In the allosteric/sensor model, the unknown protein is expected to facilitate intracellular interactions between phosphoantigens and the B30.2 domain, whereas the unknown protein in the presentation model is expected to facilitate the release of endogenous phosphoantigens and the subsequent binding to extracellular BTN3A1. It has been proposed that this hypothetical transporter only intervenes when intracellular IPP exceeds a critical threshold^40^. Our results unveils a new function of BTN3A1 by showing its contribution to extracellular IPP release in cooperation with ABCA1 and apoA-I. BTN3A1 is physically associated with ABCA1 but is not associated with apoA-I. BTN3A1 expression is not required to maintain the association between ABCA1 and IPP; it is not correlated with intracellular IPP concentrations and/or extracellular IPP release and is not affected by ZA treatment. *Btn3a1*-silenced ZA-treated DC are only marginally penalized in the ability to release extracellular IPP compared with *Abca1-*silenced or *Btn3a1/Abca1-*double silenced DC. Remarkably, ZA-treated THP-1 cells and DC^THP1^ accumulate higher amounts of intracellular IPP than ZA-treated DC (approximately 1,500 versus 900 fmol ml^−1^). In the absence of ABCA1 and despite identical BTN3A1 expression, DC^THP1^ fail to release extracellular IPP and activate Vγ9Vδ2 T cells. These data indicate that ABCA1 serves a major role in extracellular IPP release from ZA-treated DC; this function is fine-tuned by one-to-one physical interactions of ABCA1 with IPP, apoA-1 and BTN3A1. The functional hierarchy of these molecules may be very different to drive Vγ9Vδ2 T cell activation and target cell recognition once IPP has been released in the microenvironment. In the absence of ZA-treated cells, it is possible that IPP internalization in selected cell subsets and the subsequent interaction with the intracellular B30.2 domain of BTN3A1 play a more critical role than ABCA1 and apoA-I to induce Vγ9Vδ2 T cell activation.

We have deciphered two mechanisms that underlie ZA-induced ABCA1 and apoA-1 upregulation. *In vitro* chromatin immunoprecipitation (ChIP) experiments indicated that IPP induces a dose-dependent LXRα binding to the LRE of the *Abca1* promoter. The degree of DNA binding of purified LXRα incubated with 500 pM IPP was similar to the degree of DNA binding that was obtained in nuclear extracts of ZA-treated DC, the intracellular IPP levels of which range between 500 and 1,000 pM. Thus, the first mechanism is initiated by intracellular IPP accumulation due to ZA-induced FPPS inhibition, which causes LXRα activation, upregulation of *Abca1/apoA-I* transcription and increased ABCA1 and apoA-I protein expression. IPP-induced LXRα activation in our model was highly specific and cannot be reproduced with FPP or GGPP: the former had no effect, whereas GGPP downregulated LXRα activation as previously reported^25^. The antagonistic effect exerted by simvastatin confirmed the central role of IPP accumulation. By inhibiting HMGCR upstream to FPPS, simvastatin prevents ZA-induced intracellular IPP accumulation, ABCA1 and apoA-I upregulation, extracellular IPP release and Vγ9Vδ2 T cell activation. Unlike LXRα, DC express very low levels of LXRβ^41^, which remains unmodulated by ZA or simvastatin. Thus, the LXRα/LXRβ nuclear ratio is critical to fine-tune not only intracellular cholesterol concentrations in resting and activated conventional T cells to satisfy their metabolic requirements^27^ but also to regulate extracellular IPP release in DC and the subsequent activation of Vγ9Vδ2 T cells. It is also possible that phosphoantigens are internalized from the microenvironment via fluid phase endocytosis o energy-dependent mechanism^42^, especially when extracellular concentrations are much higher than intracellular concentrations, as in the supernatants from ZA-treated cells after removal of these cells. In this case, LXRα activation, ABCA1, and apoA-I upregulation may be regulated by internalization of extracellular IPP.

The second mechanism is dependent on ZA-induced inhibition of the PI3K/Akt/mTOR signalling pathway^26^,^30^,^43^. This mechanism has been described in tumour cells and associated with anti-tumour ZA activity^32^,^44^. LXRα is also regulated by the PI3K/Akt/mTOR pathway with different outcomes according to the cell type^26^,^45^. In DC, ZA-induced inhibition of the PI3K/Akt/mTOR signalling pathway increased LXRα-induced transcription of *Abca1* and *apoA-I*, which causes enhanced protein expression, increased extracellular IPP release and Vγ9Vδ2 T cell activation. Similar results were obtained by targeting the PI3K/Akt/mTOR pathway with inhibitors other than ZA, which also upregulated ABCA1 and apoA-1. These inhibitors, however, caused inferior extracellular IPP release as they do not target the mevalonate pathway and do not induce intracellular IPP accumulation.

The mechanism beyond ZA-induced PI3K/Akt/mTOR inhibition is likely due to intracellular isoprenoid deprivation instead of IPP accumulation. ZA decreases GTP-bound Ras and downstream Ras-regulated signalling pathways^29^,^30^. As activity of the PI3K/Akt/mTOR pathway is upregulated by Ras, Ras deprenylation is expected to downregulate PI3K/Akt/mTOR signalling and increase LXRα activation^28^. Simvastatin also induces intracellular isoprenoid deprivation and Ras deprenylation, but it unexpectedly restored the activity of the PI3K/Akt/mTOR signalling in ZA-treated DC. A possible explanation is that the concurrent ZA and simvastatin treatments are so stressful to induce the expression of ER-stress-related and UPR genes, which causes the recovery of PI3K/Akt/mTOR signalling^31^.

**Figure 8:**
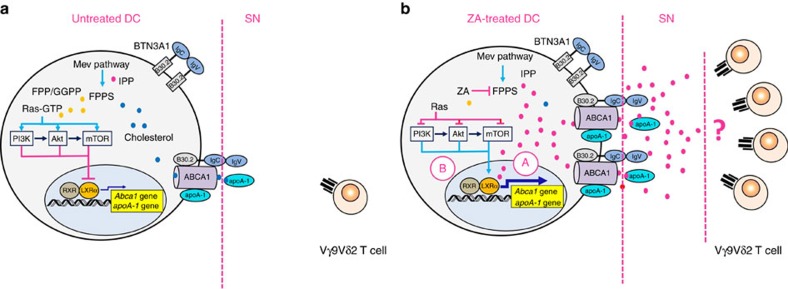
Mechanisms involved in IPP release from untreated and ZA-treated DC. (**a**): Mevalonate (Mev) pathway of untreated DC generates isoprenoids (IPP: red circles; FPP/GGPP: yellow circles) and cholesterol (blue circles). Ras is prenylated in untreated DC (Ras-GTP) and causes the activation of the PI3K/Akt/mTOR signalling pathway and suppression of LXRα transcriptional activity. ABCA1 and apoA-I expression is finalized to tune the intracellular concentrations of cholesterol and other mevalonate pathway metabolites that were generated in physiological conditions. The supernatant obtained from untreated DC is unable to induce the proliferation of Vγ9Vδ2 T cells as extracellular IPP concentrations are below a critical threshold. (**b**) Farnesyl pyrophosphate synthase (FPPS) is inhibited in ZA-treated DC, which causes intracellular IPP accumulation (red circles), and isoprenoid (yellow circles) and cholesterol (blue circles) deprivation. Intracellular IPP induces LXRα nuclear translocation (**a**), which enhances *Abca1* and *apoA-1* gene transcription and upregulation of ABCA1 and apoA-I expression. Isoprenoid deprivation concurrently causes Ras deprenylation (Ras), which relieves the inhibition operated by the PI3K/Akt/mTOR pathway on LXRα nuclear translocation (**b**). Thus, two mechanisms are operative in ZA-treated DC, which causes upregulation of ABCA1 and apoA-1 expression and extracellular IPP and apoA-I release. The supernatant from ZA-treated DC contains sufficient amounts of IPP and apoA-I to induce the activation of Vγ9Vδ2 T cells. The question mark in the right panel indicates that the mechanisms by which soluble IPP released in the supernatant from ZA-treated DC induces Vγ9Vδ2 T cell activation remain to be elucidated.

Our results indicate that ABCA1 serves a pivotal role in extracellular IPP release in cooperation with apoA-I and BTN3A1. ABCA1 is upregulated by ZA via LXRα transcriptional activation that is induced by intracellular IPP accumulation and inhibition of the PI3K/Akt/mTOR signalling pathway. The supernatants of ZA-treated DC contain sufficient IPP concentrations to induce Vγ9Vδ2 T cell activation also in the absence of DC, but the manner in which soluble IPP engages Vγ9Vδ2 T cells remains to be determined (see Fig. 8 for a schematic representation). Thus, Vγ9Vδ2 T cell activation can be regulated not only with drugs that target the mevalonate pathway, such as ZA and simvastatin but also with drugs that target the PI3K/Akt/mTOR signalling pathway. Moreover, our data credit ABCA1 with a novel immune function that is linked to the activation of Vγ9Vδ2 T cells and contributes new perspectives for understanding the relationship between lipid metabolism and immune functions.

## Methods

### Chemicals

Plasticware for cell cultures was obtained from Falcon (Becton Dickinson, Mountain View, CA). ZA was a gift from Novartis (Basel, Switzerland). Simvastatin was purchased from Calbiochem (Billerica, MA). The electrophoresis reagents were obtained from Bio-Rad Laboratories (Hercules, CA). The protein content of cell lysates was assessed with the BCA kit from Sigma Chemical Co (St Louis, MO). If not specified, all other reagents were purchased from Sigma Chemical Co.

### Cells

Peripheral blood samples were drawn from healthy blood donors; the samples were provided by the local Blood Bank (Fondazione Strumia). Peripheral blood and bone marrow samples from multiple myeloma patients and B-cell chronic lymphocytic leukaemia patients who carried mutated or unmutated immunoglobulin heavy-chain variable regions were collected after informed consent and approval by the local Institutional Review Board (Comitato Etico Interaziendale A.O.U. Città della Salute e della Scienza di Torino—A.O. Ordine Mauriziano—A.S.L. TO1) were received (DG 767/2015). After isolation on a Ficoll-Hypaque density gradient, PBMC from healthy donors were kept unfractionated or processed to purify CD14+ cells and generate different antigen-presenting cell (APC) preparations. When PBMC from chronic lymphocytic leukaemia patients contained less than 90% of CD19^+^/CD5^+^ cells, cells were purified by negative selection using a magnetic bead-activated cell sorting with a B cell Isolation Kit II (Miltenyi Biotec, Bologna, Italy). Bone marrow mononuclear cells (BMMC) from multiple myeloma and chronic lymphocytic leukaemia patients were left unfractionated to generate BMSC or were processed to purify CD138+ multiple myeloma cells. Control BMMC from healthy donors were purchased from Stem Cell Technology (Peschiera Borromeo, Italy) to generate control BMSC. SKMM1, which is a multiple myeloma cell line, THP-1 and U937 cells, which are two monocytic leukaemia cell lines, and A549 cells, which is a non-small cell lung cancer cell line, were provided by the ATCC (Manassas, VA). The standard culture medium was RPMI 1640 (Euroclone, Milano, Italy) or HAM F12 medium (Euroclone) for A549 cells, which contain 10% fetal calf serum (FCS) (Euroclone), 2 mM L-glutamine, 100 U ml^−1^ penicillin, and 100 mg ml^−1^ streptomycin. Commercial cell lines were authenticated by microsatellite analysis using the PowerPlex kit (Promega Corporation, Madison, WI; last authentication: December 2015). The cells were checked for *Mycoplasma spp.* contamination by PCR every 3 weeks, and the contaminated cells were discharged.

### Dendritic cell generation

CD14+ cells were purified using CD14 MicroBeads and LS columns (Miltenyi Biotec) and incubated for 24 h in 24-well plates at 1 × 10^6^ per ml in the presence or absence of 1 μM ZA. DC were generated as previously reported^3^. Purified CD14+ cells were cultured in a standard culture medium at 0.5–1.5 × 10^6^ cells per ml that were supplemented with GM-CSF (1000 U ml^−1^) and IL-4 (500 U ml^−1^) in flat-bottomed 24-well plates for 24 h. After 24 h, DC were left untreated (ZA-) or treated for an additional 24 h with 1 μM ZA (ZA+). To clear supernatants from any residual ZA, the DC were washed after incubation with ZA and re-plated with fresh medium for an additional 24 and 48 h (washed DC). DC^THP1^ and DC^U937^ were generated with the same procedure as previously described from THP-1 and U937 cells. The DC phenotype was verified by cytofluorimetric analysis as follows.

In selected experiments, supernatants obtained from different cell cultures were collected and saved for a subsequent quantification of intracellular IPP and extracellular IPP levels and apoA-1 and apoE levels and to investigate their ability to induce autologous Vγ9Vδ2 T cell proliferation.

### Bone marrow stromal cells generation

BMSC were generated by seeding 1 × 10^6^ BMMC/well in 24-well plates in a DMEM medium that was supplemented with 10% FCS and replaced every 3 days with fresh medium. After 4 days, non-adherent cells were washed off and adherent cells—predominantly fibroblast-like cells—were grown in DMEM medium with 10% FCS until confluence (2–3 weeks). The BMSC phenotype was determined by cytofluorimetric analysis as follows.

### Immune-phenotyping

Phenotypic characterization of DC, Vγ9Vδ2 T cells, myeloma cells and BMSC cells was performed with multicolour flow cytometry. The following anti-human monoclonal-antibody combinations were employed in the characterization: (a) Vγ9Vδ2 T cells: anti-TCR Vγ9 (clone B6, BD Pharmingen, dilution 1/50), anti-CD3 (clone BW264/56, Miltenyi Biotec, dilution 1/11); (b) DC: anti-CD80 (clone 2D10, Miltenyi Biotec, dilution 1/11), anti-CD86 (clone FM95, Miltenyi Biotec, dilution 1/11), anti-HLA-DR (clone AC122, Miltenyi Biotec, dilution 1/11); (c) myeloma cells: anti-CD138 (clone 44F9, Becton Dickinson, dilution 1/20); anti-CD38 (clone IB6, Becton Dickinson, dilution 1/20); and (d) BMSC cells: anti-CD44 (clone G44-26, Becton Dickinson, dilution 1/20), anti-CD105 (clone 266, Becton Dickinson, dilution 1/20), anti-CD11a (clone HI111, Becton Dickinson, dilution 1/20). Three- and four-colour flow cytometry were performed with the appropriate combinations of fluorescein isothiocyanate (FITC), r-phycoerythrin-(PE), Tricolour-(Tri), Peridinin Chlorophyll Protein Complex (PerCP) or allophycocyanin-(APC) conjugated antibodies, a FACScan cell sorter, and CELLQuest software (Becton Dickinson). Gating strategies are reported in the Supplementary Fig. 8.

### Vγ9Vδ2 T cell activation and proliferation

PBMC were plated for 7 days at 10^6^ cells per ml in 96-wells round-bottomed plates at 37 °C in a humidified atmosphere of 5% CO_2_ in air in the presence of 10 IU ml^−1^ IL-2 (Eurocetus, Milano, Italy) and 1 μM ZA (kindly provided by Novartis Pharma, Origgio, Italy). The standard culture medium was RPMI 1640 (Eurobio, Les Ulis, France), containing 10% FCS (Mascia Brunelli, Milano, Italy), 2 mM L-glutamine (Eurobio, Les Ulis, France), 100 U ml^−1^ penicillin (Eurobio, Les Ulis, France) and 100 mg ml^−1^ streptomycin (Eurobio, Les Ulis, France)^3^. Total counts of viable Vγ9Vδ2 T cells on day 7 were evaluated by multiplying total counts of viable cells per well, identified with the trypan blue staining assay, by the percentage of Vγ9Vδ2 T cells identified by flow cytometry^46^.

### Quantification of intracellular and extracellular IPP

After 24 h culture, 1 × 10^6^ cell per ml were incubated for another 24 h with 1 μCi of [^3^H]acetate (3,600 mCi mmol^−1^; Amersham International, Piscataway, NJ) to measure the intracellular IPP synthesis. 300 μl cells lysate or culture supernatant was diluted 1:2 into an ice-cold acetonitrile solution containing 100 mM NaVO_4_ and 250 mM NaF, and centrifuged at 1,200*g* for 5 min at 4 °C. After lyophilization under vacuum, samples were resuspended in 20 μl dimethylhexylamine and separated by thin layer chromatography (TLC; LK6D Whatmann silica gel Merck, Darmstadt, Germany), using 1:1 solution of 50% methanol/0.8 M ammonium formate, pH 7.4, containing 2 mM dimethylhexylammine as running buffer. Gels were exposed to an iodine-saturated atmosphere for 2 h, and the spot that corresponds to IPP was quantified by liquid scintillation counting (UltimaGold; PerkinElmer, Waltham, MA). According to the titration curve, the results are expressed as fmoles ml^−1^ for intracellular IPP or pmoles ml^−1^ for extracellular IPP.

### Efflux of exogenous [^14^C]-IPP

To measure the efflux of an exogenous pulse of IPP, 1 × 10^6^ cell ml^−1^ were labelled for 1 h with 0.02 μCi of [^14^C] IPP (50 mCi mmol^−1^; Amersham International),washed five times with PBS and left for 24 h in fresh medium. After this incubation time, supernatants were collected and underwent the same analytical process that was used to measure endogenously generated extracellular IPP. The amount of [^14^C]IPP that was present in the culture medium at *t*_0_ is considered to be 100%; the amount of [^14^C]IPP that was measured after incubation is expressed as a percentage relative to the amount measured at *t*_0_.

### MALDI-TOF/MS analysis

Fifty μl of the culture medium of 1 × 10^6^ CD14+ or DC were resolved by 12% SDS–PAGE, then stained using colloidal Coomassie brilliant blue (18% v-v ethanol, 15% w-v ammonium sulphate, 2% v-v phosphoric acid, 0.2% w/v Coomassie brilliant blue G-250). Gel slices from Coomassie-stained gels aligned to the recombinant human apoE or apoA-I, which were employed as standards, were excised and digested as previously described^47^. Gel slices were destained in 50% v-v acetonitrile in 5 mM ammonium bicarbonate, dried using pure acetonitrile and rehydrated for 45 min at 4 °C with digestion buffer that contains 10 ng μl^−1^ trypsin in 5 mM ammonium bicarbonate. Digestion proceeded overnight at 37 °C. The mass spectrometry analysis of peptides was performed using a matrix-assisted laser desorption ionization-time-of-flight/mass spectrometry device (MALDI micro MX; Waters, Milford, MA, USA) that was equipped with a delayed extraction unit that operated in reflectron mode. The peptide solution was prepared with equal volumes of saturated a-cyano-4-hydroxycinnamic acid solution in 40% v/v acetonitrile-0.1% v/v trifluoroacetic acid. The MALDI-TOF was calibrated with a mix of PEG (PEG 1000, 2000 and 3000 at a ratio of 1:2:2) and mass spectra were acquired in the positive-ion mode. Peak lists were generated by ProteinLynx Global Server 2.2.5 (Waters, Milford, MA, USA) using the following parameters: external calibration with lock mass using a mass of 2465.1989 Da for ACTH (adrenocorticotropic hormone), background subtract type adaptive that combined all scans, and de-isotoping with a threshold of 1%. The 25 most intense masses were employed for database searches against the SWISSPROT database using the free search program MASCOT (http://www.matrixscience.com). The following parameters were employed in the searches: taxa Homo sapiens, trypsin digest, one missed cleavage by trypsin, methionine oxidation as variable modification and allowed maximum error of 100 p.p.m.

### Western blotting

The cells were lysed in a MLB buffer (125 mM Tris-HCl, 750 mM NaCl, 1% v/v NP40, 10% v/v glycerol, 50 mM MgCl_2_, 5 mM EDTA, 25 mM NaF, 1 mM NaVO_4_, 10 μg ml^−1^ leupeptin, 10 μg ml^−1^ pepstatin, 10 μg ml^−1^ aprotinin, 1 mM phenylmethylsulphonyl fluoride, pH 7.5), sonicated and centrifuged at 13,000*g* for 10 min at 4 °C. Plasma-membrane-associated proteins were isolated by the biotinylation method, using the Surface Protein Isolation Kit (Fisher Scientific Inc., Rockford, IL)^48^. Twenty microgram of proteins from cell lysates or biotinylated extracts were subjected to western blotting and probed with the following antibodies: anti-ABCA1 (HJI, Abcam, Cambridge, UK, dilution 1/500), anti-ABCA2 (ab91571, Abcam, dilution 1/250), anti-ABCB1 (C219, Novus Biologicals, Littleton, CO, dilution 1/250), anti-ABCC1 (IU2H10, Abcam, dilution 1/100), anti-ABCC2 (M2 III-6, Abcam, dilution 1/50), anti-ABCC3 (sc-5776, Santa Cruz Biotechnology Inc., Santa Cruz, CA, dilution 1/500), anti-ABCC4 (ab77184, Abcam, dilution 1/250), anti-ABCC5 (sc-5781, Santa Cruz Biotechnology Inc., dilution 1/500), anti-ABCC6 (ab54826, Abcam, dilution 1/500), anti-ABCG1 (sc-11150, Santa Cruz Biotechnology Inc., dilution 1/1,000), anti-ABCG2 (sc-25882, Santa Cruz Biotechnology Inc., dilution 1/100), anti-ABCG4 (ab101528, Abcam, dilution 1/250), anti-ABCG5 (EPR6203, Abcam, dilution 1/500), anti-ABCG8 (ab126493, Abcam, dilution 1/500), anti-SR-BI (NB400-104, Novus Biologicals, dilution 1/1,000), anti-BTN3A1 (25221-1-AP, Proteintech, Chicago, IL, dilution 1/500), anti-apoA-I (SAB1104904, Sigma Chemical Co., dilution 1/1,000), anti-apoE (AV54283, Sigma Chemical Co., dilution 1/1,000), anti-PiT-1/SLC20A1 (ab58181, Abcam, dilution 1/200), anti-PiT-2/SLC20A2 (ab155259, Abcam, dilution 1/200), anti-NPT2A/SLC34A1 (ab151129, Abcam, dilution 1/500), anti-Na^+^/K^+^-ATPase (M7-PB-E9, Sigma Chemical Co., dilution 1/2,000), anti-Na^+^/H^+^ exchanger 1/NHE1 (sc-136239, Santa Cruz Biotechnology Inc., dilution 1/500), anti-Cl^-^/HCO_3_^-^ transporter/SLC4A1 (EPR1426, Abcam, dilution 1/1,000), anti-phospho(Ser473)Akt (6F5, Millipore, Billerica, MA, dilution 1/1,000), anti-Akt (SKB1, Millipore, dilution 1/500), anti-phospho(Thr389)-p70 S6K (#9205, Cell Signalling Technology, Danvers, MA, dilution 1/1,000), anti-phospho(Thr421/Ser424)-p70 S6K (#9204, Cell Signalling Technology, dilution 1/1,000), and anti-p70 S6K (#9202, Cell Signalling Technology, dilution 1/1,000). Anti-β-tubulin (sc-5274, Santa Cruz Biotechnology Inc., dilution 1/1,000) and anti-pancadherin (sc-1499, Santa Cruz Biotechnology Inc., dilution 1/500) were employed as a control of equal protein loading in whole cell and plasma-membrane extracts, followed by a peroxidase-conjugated secondary antibody. The proteins were detected by enhanced chemiluminescence (Bio-Rad Laboratories).

In the co-immunoprecipitation assays, 100 μg of proteins from biotinylated plasma-membrane extracts, which were prepared as previously described, were immunoprecipitated with the anti-BTN3A1 antibody, using the PureProteome protein A and protein G Magnetic Beads (Millipore). The immunoprecipitated proteins were separated by SDS–PAGE and probed with the anti-ABCA1 or apoA-I antibodies. An anti-pancadherin antibody was employed as the control for equal plasma-membrane protein loading.

To evaluate LXRα, LXRβ and RXR nuclear translocations, nuclear extracts were prepared using the Nuclear Extract kit (Active Motif, La Hulpe, Belgium). Ten microgram of nuclear proteins were resolved by SDS–PAGE and probed with anti-LXRα (61175, Active Motif, dilution 1/500), anti-LXRβ (ABN65, Millipore, dilution 1/500), anti-RXR (ab24363, Abcam, dilution 1/2,000) antibodies or with an anti-TFIID/TATA box-binding protein (TBP) antibody (58C9, Santa Cruz Biotechnology Inc., dilution 1/250) as the equal protein loading control.

The images of uncropped gels reported in main figures and Supplementary Figures are shown in Supplementary Figs 9 and 10.

### Abca1 and Btn3a1 silencing

Two × 10^5^ DC were transfected with Accell Human siRNA ABCA1, Accell Human BTN3A1 siRNA or 19–25 nucleotide non-targeting scrambled siRNAs (from Thermo Scientific Open Biosystems, Waltham, MA) per the manufacturer's instructions. The efficacy of silencing was verified by western blotting, as previously described.

### Proximity ligation assay

The presence of ABCA1-BTN3A1 interactions was assessed with the PLA method using the DuoLink In Situ kit (Sigma Chemical Co.) per the manufacturer's instructions. A total of 1 × 10^4^ cells were seeded on sterile coverglass, washed with PBS, fixed for 10 min at room temperature with 4% v/v paraformaldehyde, washed twice with PBS, and incubated 30 min at 37 °C in a humidified chamber with 40 μl of the kit-provided Blocking solution. A mouse anti-human ABCA1 (HJI, Abcam) and rabbit anti-human BTN3A1 (HPA012565, Sigma Chemical Co.) antibodies were added at 1:50 final dilution. Samples were maintained at 4 °C overnight. Slides were washed once with the Wash buffer A for 5 min at room temperature, and incubated 1 h at 37 °C with the respective PLA probes diluted 1:5 into 40 μl diluent antibody solution. Samples were washed twice with wash buffer A for 5 min at room temperature and incubated for 30 min at 37 °C with 40 μl ligation solution diluted 1:5 into RNAse/DNAse-free water and containing 25 mU ligase. After two washes at the rate of 2 min per each at room temperature, amplification was performed by adding 40 μl amplification solution that was diluted 1:5 into RNAse/DNAse-free water and contained 125 mU polymerase. Samples were maintained for 2 h at 37 °C, washed twice with undiluted wash buffer B for 10 min, once with wash buffer B, diluted 1:100 for 1 min, and mounted with the DuoLink In Situ Mounting medium, which contained 4′,5-diamidino-2 phenylindole dyhydrochloride (DAPI) to counterstain cells' nuclei. Cells were examined using a Leica TCS SP2 AOP confocal laser-scanning microscope (Leica Microsystem, Wetzlar, Germany). A minimum of five fields were examined for each experimental condition.

### Crosslinking assay

Crosslinking of apoA-I to cell surface proteins was performed as previously reported^20^. After labelling for 2 h at 37 °C with 5 μg ml^−1^ [^125^I]-apoA-I and chemically crosslinking with dithiobis(succinimidyl propionate) (DSP), cells were lysed and subjected to immunoprecipitation with an anti-apoA-I antibody (SAB1104904, Sigma Chemical Co., dilution 1/100). Samples were resolved by SDS–PAGE, and blotted proteins were detected by autoradiography (Kodak BioMax MR-1, PerkinElmer).

### IPP-ABCA1 interaction

IPP-ABCA1 interaction was measured in the cells labelled with 1 μM [^14^C]-IPP for 1 h. After extensively washing (five washing steps of 5 min with PBS) to remove any traces of residual [^14^C]-IPP in the extracellular medium, the cells were grown in fresh medium for 1 h and fixed with DSP. The surface proteins were isolated with the Surface Protein Isolation Kit (Fisher Scientific Inc. A total of 500 μg of surface proteins were subjected to immunoprecipitation with an anti-ABCA1 antibody (HJI, Abcam, dilution 1/50). As an internal control, cells were incubated 5 min with 2 mM dithiothreitol, and the biotinylated extracts were subjected to limited digestion with 10 μg ml^−1^trypsin, as reported^21^,^22^. Immunopurified ABCA1 was resolved by SDS–PAGE, and run in duplicate on the same gel. One part of the gel was probed with the anti-ABCBA antibody; the second part was dried and subjected to autoradiography to visualize the [^14^C]-IPP signal.

### Quantitative real time-PCR and PCR array

Total RNA was reverse-transcribed using the iScript cDNA Synthesis Kit (Bio-Rad Laboratories). The qRT-PCR was performed with the IQ SYBR Green Supermix (Bio-Rad). The same cDNA preparation was employed to quantify the genes of interest and the housekeeping gene *S14*. The primer sequences, which were designed using the qPrimerDepot database (http://primerdepot.nci.nih.gov/), were as follows: for *Abca1*: 5′-CAGAGCTCACAGCAGGGAC-3′; 5′-CTTCTCCGGAAGGCTTGTC-3′; for *apoA-I*: 5′-CCCAGTTGTCAAGGAGCTTT-3′, 5′-TGGATGTGCTCAAAGACAGC-3′; for *S14*: 5′-CGAGGCTGATGACCTGTTCT-3′; 5′-GCCCTCTCCCACTCTCTCTT-3′. The relative quantification was performed by comparing each PCR product with the housekeeping PCR product *S14*, using the Bio-Rad Software Gene Expression Quantitation (Bio-Rad). PCR arrays were generated using 1 μg cDNA and Human Unfolded Protein Response Plus RT^2^ Profiler* PCR* Array (Bio-Rad Laboratories) according to the manufacturer's instructions. Data analysis was performed using the PrimePCR Analysis Software (Bio-Rad Laboratories).

### Chromatin Immunoprecipitation

ChIP samples were prepared as described by Campia *et al*.^49^, using a ChIP-tested anti-LXRα antibody (61175, Active Motif, dilution 1/50). The putative Liver X Receptor Response Element (LRE) sites on *Abca1* and *apoA-I* human promoters were validated with the Matinspector Software (https://www.genomatix.de/matinspector.html). Primer sequences were as follows: for *Abca1* promoter: 5′-GGAGAGCACAGGCTTTGACC-3′; 5′-CTCTCGCGCAATTACGGG-3′; for *apoA-I* promoter: 5′-AACTGCCCACACACTCCCAT-3′; 5′-TCCTTCTCGCAGTCTCTAAGCA-3′. Primers used as negative internal controls for a nonspecific 10 000 bp upstream sequence, were as follows: 5′-GTGGTGCCTGAGGAAGAGAG-3′ and 5′-GCAACAAGTAGGCACAAGCA-3′. The immunoprecipitated products were amplified by qRT-PCR.

In *in vitro* ChIP experiments, total DNA from 1 × 10^6^ DC was extracted using the GenElute Mammalian Genomic DNA Miniprep Kit (Sigma Chemical Co.), sonicated, incubated with 1 ng of recombinant human LXRα protein (Abcam), immunoprecipitated with the anti-LXRα antibody and processed as previously described.

### Activity of plasma-membrane-associated transporter and channels

Plasma-membrane vesicles enriched for ATP-binding cassette transporters were prepared as detailed elsewhere with minor modifications^50^. Cells were washed with Ringer's solution (148.7 mM NaCl, 2.55 mM K_2_HPO_4_, 0.45 mM KH_2_PO_4_, 1.2 mM MgSO_4_; pH 7.4), lysed on crushed ice with lysis buffer (10 mM Hepes/Tris, 5 mM EDTA, 5 mM EGTA, 2 mM dithiothreitol; pH 7.4) supplemented with 2 mM phenylmethylsulfonyl fluoride, 1 mM aprotinin, 10 μg ml^−1^ pepstatin, 10 μg ml^−1^ leupeptin, and subjected to nitrogen cavitation at 1200, p.s.i for 20 min. Samples were centrifuged at 300*g* for 10 min, diluted 1:4 in the pre-centrifugation buffer (10 mM Tris/HCl, 25 mM sucrose; pH 7.5), overlaid on a sucrose cushion (10 mM Tris/HCl, 35% w/v sucrose, 1 mM EDTA; pH 7.5) and centrifuged at 14,000*g* for 10 min. The interface was collected, diluted 1:5 in the centrifugation buffer (10 mM Tris/HCl, 250 mM sucrose; pH 7.5) and subjected to a third centrifugation at 100,000 × *g* for 45 min. The vesicle pellet was resuspended in 0.5 ml centrifugation buffer and stored at −80 °C until the use, after the quantification of the protein content. Proteins were extracted by non-denaturing immunoprecipitation using the previously indicated antibodies. The ATPase activity of immunopurified ABCA1, ABCA2, ABCB1, ABCC1, ABCC3, ABCC4, ABCG1, ABCG2 and ABCG4 was measured with a spectrophotometric method^51^. Samples (containing 25 μg protein) were incubated for 30 min at 37 °C with 50 μl of the reaction mix (25 mM Tris/HCl, 3 mM ATP, 50 mM KCl, 2.5 mM MgSO4, 3 mM dithiothreitol, 0.5 mM EGTA, 2 mM ouabain, 3 mM NaN3; pH 7.0). In each set of experiments, a blank containing 0.5 mM Na_3_VO_4_ was included^52^. The reaction was stopped by adding 0.2 ml ice-cold stopping buffer (0.2% w/v ammonium molybdate, 1.3% v/v H_2_SO_4_, 0.9% w/v SDS, 2.3% w/v trichloroacetic acid, 1% w/v ascorbic acid). After a 30 min incubation at room temperature, the absorbance of the phosphate hydrolyzed from ATP was measured at 620 nm, using a Packard EL340 microplate reader (Bio-Tek Instruments, Winooski, MA). The absorbance was converted into μmol hydrolyzed phosphate per min per mg proteins, according to the titration curve previously prepared. The activity of PiT-1/SLC20A1 and PiT-2/SLC20A2 (ref. 50), and Na^+^/K^+^-ATPase^53^ were spectrophotometrically measured. The results are expressed as nmoles of hydrolysed phosphate per min per mg proteins according to the previously prepared titration curve. The NHE1 and SLC4A1 activities were fluorimetrically measured^54^,^55^, and the results are expressed as ΔpHi/min and arbitrary units, respectively.

### Statistical analysis

The results are expressed as the mean±s.e.m. Differences between the groups were evaluated with a one-way analysis of variance, a Wilcoxon–Mann–Whitney non-parametric test for paired or unpaired samples as appropriate and was considered to be statistically significant for *P* values <0.05. Correlation analyses were performed with the non-parametric Spearman Rank Order test with a cutoff *P* value <0.05. The GraphPad software was used for the statistical analyses; its symbology has been adopted to define the statistical significance (ns=*P*>0.05; *P<0.05; **P<0.01; ***P<0.001; ****P<0.0001). The sample size was calculated with the G*Power software (www.gpower.hhu.de), with the setting *P*<0.05 as the significance level and 0.80 as the power of the study.

### Data availability

The authors declare that all data supporting the findings of this study are available within the paper and its Supplementary Information files.

## Additional information

**How to cite this article:** Castella, B. *et al*. The ATP-binding cassette transporter A1 regulates phosphoantigen release and Vγ9Vδ2 T cell activation by dendritic cells. *Nat. Commun.* **8,** 15663 doi: 10.1038/ncomms15663 (2017).

**Publisher's note:** Springer Nature remains neutral with regard to jurisdictional claims in published maps and institutional affiliations.

## Supplementary Material

Supplementary Information Supplementary Figures and Supplementary Table

Supplementary Data 1 Expression of UPR genes after ZA and simvastatin treatment. The expression of ER stress-related genes was measured in untreated DC and DC treated with ZA (1 μM) and simvastatin (SIMV; 1 μM) for 24 h. Tunicamycin (Tun) (1 μM) was included as an inducer of ER stress. Total mRNA was extracted, retro-transcribed and amplified with specific primers for ER stress-related genes, which are contained in the Human Unfolded Protein Response Plus RT^2^ Profiler(tm) PCR Array (Bio-Rad Laboratories). Data analysis was performed using the PrimePCR(tm) Analysis Software (Bio-Rad Laboratories). Fold-Change (2^(- Delta Delta Ct)) is the normalized gene expression (2^(- Delta Ct)) in treated DC, which divided the normalized gene expression (2^(- Delta Ct)) in untreated DC (n= 4), where Ct is the threshold cycle in qRT-PCR. Fold-change values greater than one indicate up-regulation, whereas fold-change values less than one indicate down-regulation. The P values are calculated based on Student's t test of the replicate 2^(- Delta Ct) values for each gene. P < 0.05 was considered to be significant. ERAD: ER-associated degradation; ERQC: ER-quality control.

## References

[b1] Gober H. J. . Human T cell receptor gammadelta cells recognize endogenous mevalonate metabolites in tumor cells. J. Exp. Med. 197, 163–168 (2003). 12538656 10.1084/jem.20021500 PMC2193814

[b2] Roelofs A. J., Thompson K., Gordon S. & Rogers M. J. Molecular mechanisms of action of bisphosphonates: current status. Clin. Cancer Res. 12, 6222s–6230s (2006). 17062705 10.1158/1078-0432.CCR-06-0843

[b3] Castella B. . Immune modulation by zoledronic acid in human myeloma: an advantageous cross talk between Vγ9Vδ2 T cells, αβ CD8+ T cells, regulatory T cells, and dendritic cells. J. Immunol. 187, 1578–1590 (2011). 21753152 10.4049/jimmunol.1002514

[b4] Harly C., Peigné C. M. & Scotet E. Molecules and mechanisms implicated in the peculiar antigenic activation process of human Vγ9Vδ2 T Cells. Front. Immunol. 5, 657 (2015). 25601861 10.3389/fimmu.2014.00657 PMC4283718

[b5] Fiore F. . Enhanced ability of dendritic cells to stimulate innate and adaptive immunity on short-term incubation with zoledronic acid. Blood 110, 921–927 (2007). 17403919 10.1182/blood-2006-09-044321

[b6] Harly C. . Key implication of CD277/butyrophilin-3 (BTN3A) in cellular stress sensing by a major human γδ T cell subset. Blood 120, 2269–2279 (2012). 22767497 10.1182/blood-2012-05-430470 PMC3679641

[b7] Vavassori S. . Butyrophilin 3A1 binds phosphorylated antigens and stimulates human γδ T cells. Nat. Immunol. 14, 908–916 (2013). 23872678 10.1038/ni.2665

[b8] Gu S., Nawrocka W. & Adams E. J. Sensing of pyrophosphate metabolites by Vγ9Vδ2 T Cells. Front Immunol 5, 688 (2015). 25657647 10.3389/fimmu.2014.00688 PMC4303140

[b9] Palakodeti A. . The molecular basis for modulation of human Vγ9Vδ2 T cell responses by CD277/butyrophilin-3 (BTN3A)-specific antibodies. J. Biol. Chem. 287, 32780–32790 (2012). 22846996 10.1074/jbc.M112.384354 PMC3463320

[b10] Vantourout P. . Specific requirements for Vgamma9Vdelta2 T cell stimulation by a natural adenylated phosphoantigen. J. Immunol. 183, 3848–3857 (2009). 19710470 10.4049/jimmunol.0901085 PMC2809082

[b11] Scotet E. . Tumor recognition following Vgamma9Vdelta2 T cell receptor interactions with a surface F1-ATPase-related structure and apolipoprotein A-I. Immunity 22, 71–80 (2005). 15664160 10.1016/j.immuni.2004.11.012

[b12] Mookerjee-Basu J. . F1-adenosine triphosphatase displays properties characteristic of an antigen presentation molecule for Vgamma9Vdelta2 T cells. J. Immunol. 184, 6920–6982 (2010). 20483757 10.4049/jimmunol.0904024

[b13] Champagne E., Martinez L. O., Vantourout P., Collet X. & Barbaras R. Role of apolipoproteins in gammadelta and NKT cell-mediated innate immunity. Immunol. Res. 33, 241–255 (2005). 16462001 10.1385/ir:33:3:241

[b14] Hafiane A. & Genest J. HDL, atherosclerosis, and emerging therapies. Cholesterol 2013, 891403 (2013). 23781332 10.1155/2013/891403 PMC3678415

[b15] Shichiri M. . ATP-binding cassette transporter A1 is involved in hepatic alpha-tocopherol secretion. J. Nutr. Biochem. 21, 451–456 (2010). 19427182 10.1016/j.jnutbio.2009.02.002

[b16] Berges C. . A cell line model for the differentiation of human dendritic cells. Biochem. Biophys. Res. Commun. 333, 896–907 (2005). 15963458 10.1016/j.bbrc.2005.05.171

[b17] Chan W. K., Cheung C. C., Law H. K., Lau Y. L. & Chan G. C. Ganoderma lucidum polysaccharides can induce human monocytic leukemia cells into dendritic cells with immuno-stimulatory function. J. Hematol. Oncol. 1, 9 (2008). 18644156 10.1186/1756-8722-1-9 PMC2517069

[b18] dos Santos G. G. . Progress on the development of human *in vitro* dendritic cell based assays for assessment of the sensitizing potential of a compound. Toxicol. Appl. Pharmacol. 236, 372–382 (2009). 19232364 10.1016/j.taap.2009.02.004

[b19] Yamamoto S. . Pharmacologic suppression of hepatic ATP-binding cassette transporter 1 activity in mice reduces high-density lipoprotein cholesterol levels but promotes reverse cholesterol transport. Circulation 124, 1382–1390 (2011). 21859969 10.1161/CIRCULATIONAHA.110.009704 PMC3323112

[b20] Vedhachalam C. . ABCA1-induced cell surface binding sites for apoA-I. Arterioscler. Thromb. Vasc. Biol. 27, 1603–1609 (2007). 17478755 10.1161/ATVBAHA.107.145789

[b21] Takahashi K., Kimura Y., Kioka N., Matsuo M. & Ueda K. Purification and ATPase activity of human ABCA1. J. Biol. Chem. 281, 10760–10768 (2006). 16500904 10.1074/jbc.M513783200

[b22] Hozoji M., Kimura Y., Kioka N. & Ueda K. Formation of two intramolecular disulfide bonds is necessary for apoA-I-dependent cholesterol efflux mediated by ABCA1. J. Biol. Chem. 284, 11293–11300 (2009). 19258317 10.1074/jbc.M900580200 PMC2670134

[b23] Costet P., Luo Y., Wang N. & Tall A. R. Sterol-dependent transactivation of the ABC1 promoter by the liver X receptor/retinoid X receptor. J. Biol. Chem. 275, 28240–28245 (2000). 10858438 10.1074/jbc.M003337200

[b24] Lee J., Tauscher A., Seo D. W., Oram J. F. & Kuver R. Cultured gallbladder epithelial cells synthesize apolipoproteins A-I and E. Am. J. Physiol. Gastrointest. Liver Physiol. 285, G630–G641 (2003). 12773300 10.1152/ajpgi.00101.2003

[b25] Gan J. . Dual mechanisms of ABCA1 regulation by geranylgeranyl pyrophosphate. J Biol Chem. 276, 48702–48708 (2001). 11641412 10.1074/jbc.M109402200

[b26] Dong F., Mo Z., Eid W., Courtney K. C. & Zha X. Akt inhibition promotes ABCA1-mediated cholesterol efflux to apoA-I through suppressing mTORC1. PLoS One 9, e113789 (2014). 25415591 10.1371/journal.pone.0113789 PMC4240609

[b27] Thurnher M. & Gruenbacher G. T lymphocyte regulation by mevalonate metabolism. Sci. Signal. 8, re4 (2015). 25829448 10.1126/scisignal.2005970

[b28] Okkenhaug K. Signaling by the phosphoinositide 3-kinase family in immune cells. Annu. Rev. Immunol. 31, 675–704 (2013). 23330955 10.1146/annurev-immunol-032712-095946 PMC4516760

[b29] Riganti C. . Zoledronic acid restores doxorubicin chemosensitivity and immunogenic cell death in multidrug-resistant human cancer cells. PLoS ONE 8, e60975 (2013). 23593363 10.1371/journal.pone.0060975 PMC3625183

[b30] Moriceau G. . Zoledronic acid potentiates mTOR inhibition and abolishes the resistance of osteosarcoma cells to RAD001 (Everolimus): pivotal role of the prenylation process. Cancer Res. 70, 10329–10334 (2010). 20971812 10.1158/0008-5472.CAN-10-0578 PMC3097388

[b31] Wang J., Yang X. & Zhang J. Bridges between mitochondrial oxidative stress, ER stress and mTOR signaling in pancreatic β cells. Cell Signal 28, 1099–1104 (2016). 27185188 10.1016/j.cellsig.2016.05.007

[b32] Lan Y. C. . Zoledronic acid-induced cytotoxicity through endoplasmic reticulum stress triggered REDD1-mTOR pathway in breast cancer cells. Anticancer Res. 33, 3807–3814 (2013). 24023313

[b33] Ghavami S. . Apoptosis, autophagy and ER stress in mevalonate cascade inhibition-induced cell death of human atrial fibroblasts. Cell Death Dis. 3, e330 (2012). 22717585 10.1038/cddis.2012.61 PMC3388233

[b34] Tanaka Y. . Natural and synthetic non-peptide antigens recognized by human gamma delta T cells. Nature 375, 155–158 (1995). 7753173 10.1038/375155a0

[b35] Gruenbacher G. . Stress-related and homeostatic cytokines regulate Vγ9Vδ2 T cell surveillance of mevalonate metabolism. Oncoimmunology 3, e953410 (2014). 25960933 10.4161/21624011.2014.953410 PMC4368140

[b36] Rhodes D. A. . Activation of human γδ T cells by cytosolic interactions of BTN3A1 with soluble phosphoantigens and the cytoskeletal adaptor periplakin. J Immunol. 194, 2390–2398 (2015). 25637025 10.4049/jimmunol.1401064 PMC4337483

[b37] Sandstrom A. . The intracellular B30.2 domain of butyrophilin 3A1 binds phosphoantigens to mediate activation of human Vγ9Vδ2 T cells. Immunity 40, 490–500 (2014). 24703779 10.1016/j.immuni.2014.03.003 PMC4028361

[b38] Riaño F. . Vγ9Vδ2 TCR-activation by phosphorylated antigens requires butyrophilin 3 A1 (BTN3A1) and additional genes on human chromosome 6. Eur. J. Immunol. 44, 2571–2576 (2014). 24890657 10.1002/eji.201444712

[b39] Wang H. & Morita C. T. Sensor function for butyrophilin 3A1 in prenyl pyrophosphate stimulation of human Vγ2Vδ2 T Cells. J. Immunol. 195, 4583–4594 (2015). 26475929 10.4049/jimmunol.1500314 PMC4848273

[b40] De Libero G., Lau S. Y. & Mori L. Phosphoantigen presentation to TCR γδ cells, a cnundrum getting less gray zones. Front. Immunol. 5, 679 (2015). 25642230 10.3389/fimmu.2014.00679 PMC4295553

[b41] Geyeregger R. . Liver X receptors regulate dendritic cell phenotype and function through blocked induction of the actin-bundling protein fascin. Blood 109, 4288–4295 (2007). 17255360 10.1182/blood-2006-08-043422

[b42] Kilcollins A. M., Li J., Hsiao C. H. & Wiemer A. J. HMBPP analog prodrugs bypass energy-dependent uptake to promote efficient BTN3A1-mediated malignant cell lysis by Vgamma9Vdelta2 T lymphocyte effectors. J. Immunol. 197, 419–428 (2016). 27271567 10.4049/jimmunol.1501833 PMC4935553

[b43] Polakiewicz R. D., Schieferl S. M., Gingras A. C., Sonenberg N. & Comb M. J. mu-Opioid receptor activates signaling pathways implicated in cell survival and translational control. J. Biol. Chem. 273, 23534–23541 (1998). 9722592 10.1074/jbc.273.36.23534

[b44] Li S. & De Souza P. Ras isoprenylation and pAkt inhibition by zoledronic acid and fluvastatin enhances paclitaxel activity in T24 bladder cancer cells. Cancers (Basel) 3, 662–674 (2011). 24212635 10.3390/cancers3010662 PMC3756383

[b45] Hwahng S. H., Ki S. H., Bae E. J., Kim H. E. & Kim S. G. Role of adenosine monophosphate-activated protein kinase-p70 ribosomal S6 kinase-1 pathway in repression of liver X receptor-alpha-dependent lipogenic gene induction and hepatic steatosis by a novel class of dithiolethiones. Hepatology 49, 1913–1925 (2009). 19378344 10.1002/hep.22887

[b46] Mariani S. . Effector gammadelta T cells and tumor cells as immune targets of zoledronic acid in multiple myeloma. Leukemia 19, 664–670 (2005). 15744346 10.1038/sj.leu.2403693

[b47] Mandili G. . Characterization of the protein ubiquitination response induced by Doxorubicin. FEBS J. 279, 2182–2191 (2012). 22536838 10.1111/j.1742-4658.2012.08602.x

[b48] De Boo S. . iNOS activity is necessary for the cytotoxic and immunogenic effects of doxorubicin in human colon cancer cells. Mol. Cancer 8, 108 (2009). 19925669 10.1186/1476-4598-8-108 PMC2785770

[b49] Campia I. . Digoxin and ouabain induce the efflux of cholesterol via liver X receptor signalling and the synthesis of ATP in cardiomyocytes. Biochem. J. 447, 301–311 (2012). 22845468 10.1042/BJ20120200

[b50] Litman T., Zeuthen T., Skovsgaard T. & Stein W. D. Competitive, non-competitive and cooperative interactions between substrates of P-glycoprotein as measured by its ATPase activity. Biochim. Biophys. Acta. 1361, 169–176 (1997). 9300798 10.1016/s0925-4439(97)00027-6

[b51] Kopecka J. . Insights in the chemical components of liposomes responsible for P-glycoprotein inhibition. Nanomedicine 10, 77–87 (2014). 23850894 10.1016/j.nano.2013.06.013

[b52] Beck L. . The phosphate transporter PiT1 (Slc20a1) revealed as a new essential gene for mouse liver development. PLoS ONE 5, e9148 (2010). 20161774 10.1371/journal.pone.0009148 PMC2818845

[b53] Bełtowski J., Jamroz-Wiśniewska A., Nazar J. & Wójcicka G. Spectrophotometric assay of renal ouabain-resistant Na+-ATPase and its regulation by leptin and dietary-induced obesity. Acta Biochim. Pol. 51, 1003–1014 (2004). 15625572

[b54] Miraglia E. . Na1/H1 exchanger activity is increased in doxorubicin-resistant human colon cancer cells and its modulation modifies the sensitivity of the cells to doxorubicin. Int J Cancer. 115, 924–929 (2005). 15729714 10.1002/ijc.20959

[b55] Nagarajan S. . Mechanical perturbations trigger endothelial nitric oxide synthase activity in human red blood cells. Sci. Rep. 6, 26935 (2016). 27345770 10.1038/srep26935 PMC4921846

