# Supplementary material for: The ATP-binding cassette transporter A1 regulates phosphoantigen release and Vγ9Vδ2 T cell activation by dendritic cells
Source: Nat Commun. 2017 Jun 5;8:15663. doi: 10.1038/ncomms15663 (PMC5465356; doi:10.1038/ncomms15663)
Supplement: Supplementary Information — Supplementary Figures and Supplementary Table [file ncomms15663-s1.pdf]

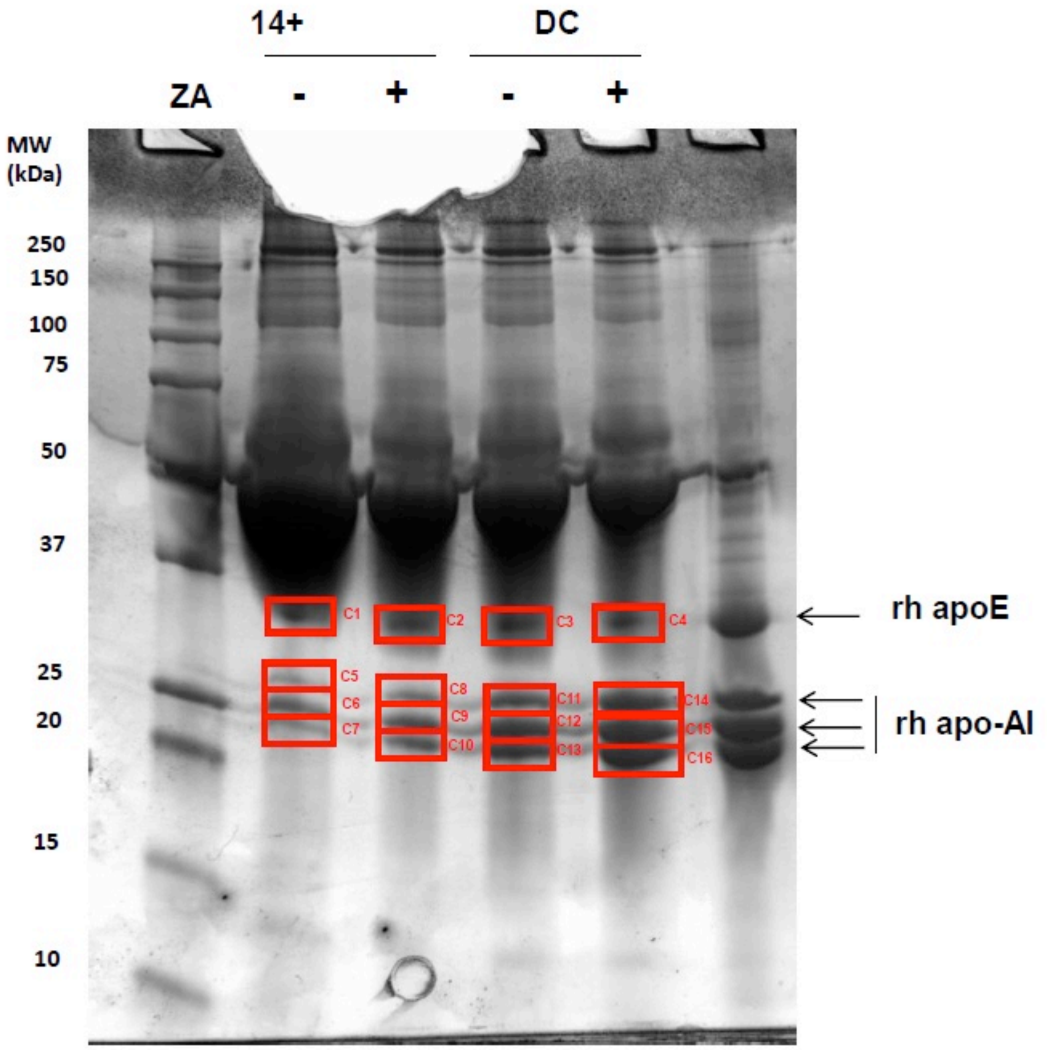

Supplementary Figure 1. Supernatants electrophoresis from CD14+ and dendritic cells. Supernatants were resolved by SDS-PAGE and stained with Coomassie brilliant blue. One µg/ml recombinant human (rh) apo-E or apoA-I were employed as standards. Bands were cut and analysed by MALDI-TOF/MS (refer to Supplementary Table 1 for details) are highlighted.

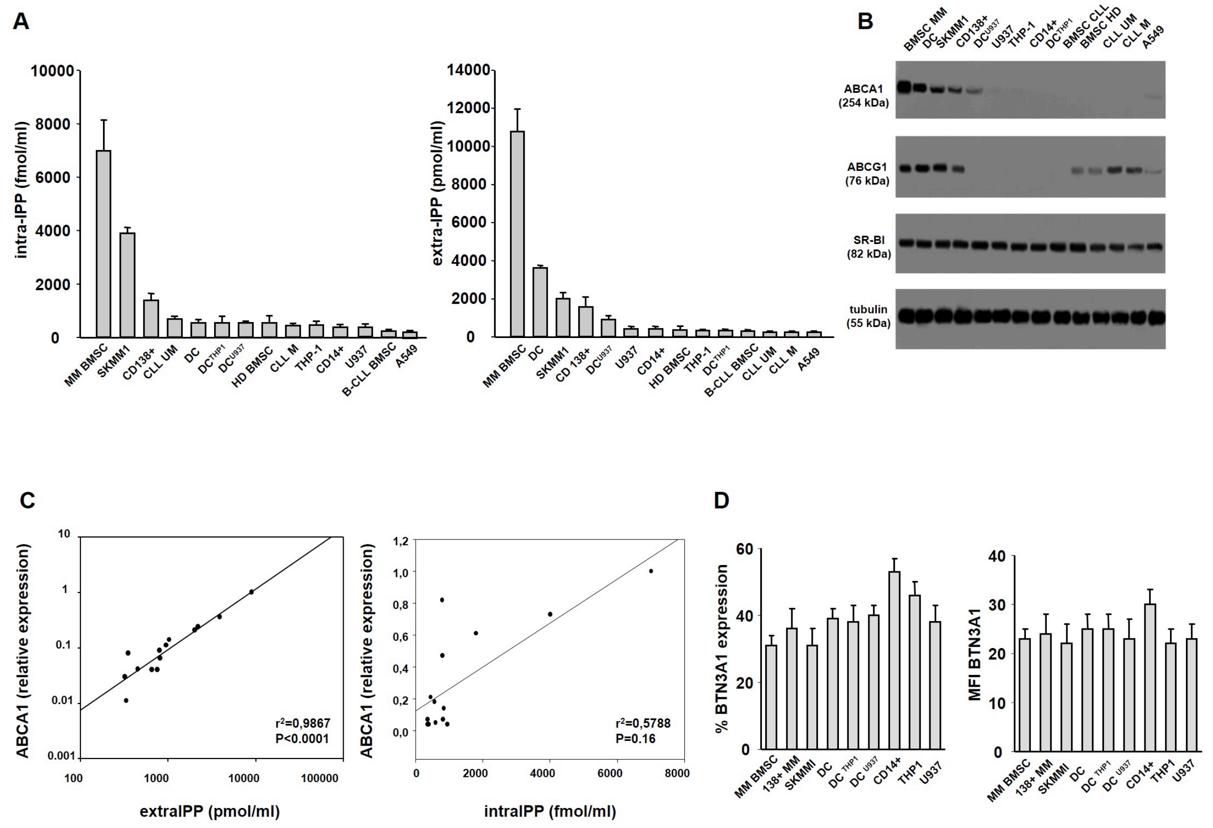

Supplementary Figure 2. Correlation between extracellular IPP release and ABCA1. Intracellular and extracellular IPP levels **(A)** and ABCA1, ABCG1 and SR-BI expression **(B)** were investigated in CD14+ cells and DC (n=11) from healthy donors; THP-1 and U937 cells and corresponding DC subsets (DC^{THP1}, DC^{U937}) (n=3); primary CD138+ cells isolated from the bone marrow of myeloma patients (CD138+ MM) (n=3); the human multiple myeloma SKMM1 cell line (n = 3); primary B-cell chronic lymphocytic leukaemia (B-CLL) cells from IGHV unmutated (UM) and mutated (M) patients (n=15); bone marrow stromal cells (BMSC) derived from healthy donors (HD BMSC) (n=8), MM patients (MM BMSC) (n = 12), B-CLL patients (B-CLL BMSC) (n=6); and the non-small cell lung cancer A549 cell line, which was included as a non-haematological cell line. **A)** Intracellular and extracellular IPP were measured after [^{3}H]-acetate radiolabelling. Cells are ranked according to the amount of extracellular IPP release. **B)** Western blot analysis of ABCA1, ABCG1, and SR-BI in whole cell lysates. β-tubulin was employed as a control of equal protein loading. Cells are ranked according to ABCA1 expression levels. The blots are representative of 1 out of 3 experiments. **C)** Correlations between extracellular and intracellular IPP and ABCA1 expression. The mean band density of ABCA1 (panel B) was calculated using ImageJ software (http://www.rsb.info.nih.gov/ij/), expressed as arbitrary units (where the band density in CD14+ cells was set to 1) and plotted against extracellular (left panel) or intracellular (right panel) IPP levels in each cell sample. The R^2 coefficient was calculated using Fig.P software (Fig.P Software Inc., Hamilton, Canada). **D)** Cytofluorimetric analysis of cell surface BTN3A1 expression in selected cell types from panels A-B. No statistically significant differences were observed in terms of percentage and mean fluorescence intensity (MFI).

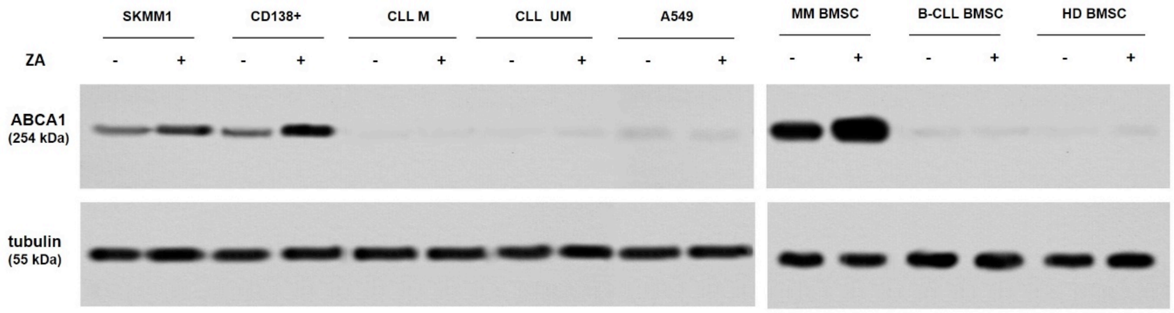

Supplementary Figure 3. ZA-induced ABCA1 upregulation in different cells.
The multiple myeloma SKMM1 cell line, primary myeloma cells (CD138+ MM), primary CLL cells from patients with mutated (M) and unmutated (UM) chronic lymphocytic leukaemia (CLL), the non-small cell lung cancer A549 cell line, and bone marrow stromal cells derived from MM patients (MM BMSC), CLL patients (CLL BMSC) or healthy donors (HD BMSC) were incubated for 24 h in the absence (-) or in the presence (+) of 1 μM ZA. Whole cell lysates were analysed for ABCA1 expression by Western blotting. ZA increased ABCA1 expression on cells that already expressed ABCA1 in baseline conditions. β-tubulin was employed to check the equal protein loading per lane. The blots are representative of 1 of 3 experiments.

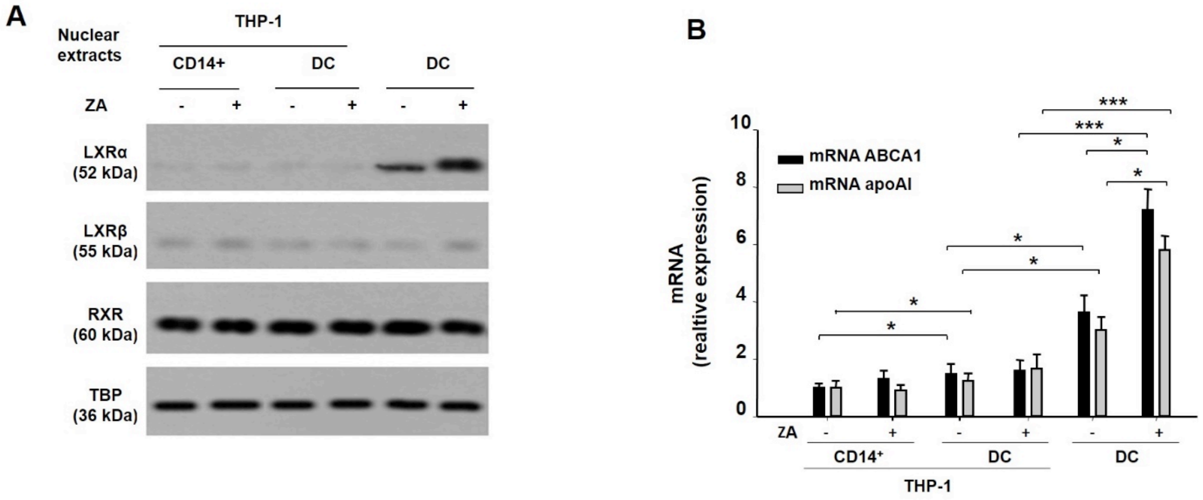

Supplementary Figure 4. LXRα activity, ABCA1 and apoA-I expression in THP-1 cells. THP-1 cells and DC^{THP1} were incubated for 24 h in the absence (-) or presence (+) of 1 μM ZA. DC generated from healthy donors (DC) were employed as internal positive control. **A)** LXRα, LXRβ and RXR levels were measured by Western blotting in nuclear extracts. In contrast to normal DC, THP-1 cells exhibited a low LXRα/LXRβ ratio, which were not modified by ZA. TBP served as a control for equal protein loading. The blots are representative of 1 of 3 experiments. **B)** *Abca1* and *apoA-I* mRNA levels. Total mRNA was extracted, retro-transcribed and amplified by qRT-PCR. THP-1 cells have significantly lower levels of *Abca1* and *apoA-I* mRNA than DC irrespective of ZA treatment. The bars represent the mean ± s.e.m. of 3 experiments ($^{*}P < 0.05$, $^{***}P < 0.001$; ANOVA).

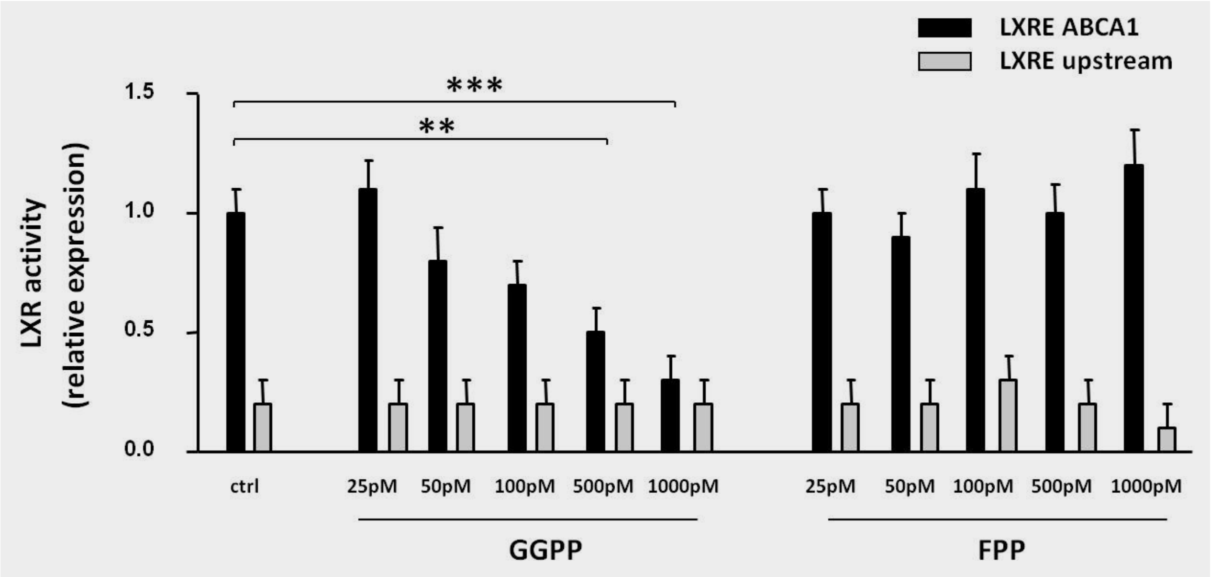

Supplementary Figure 5. Effect of GGPP and FPP on LXRα transcriptional activity. Evaluation of LXRα binding to LRE sequences in the *Abca1* promoter by ChIP assay. Genomic DNA extracted from DC and recombinant human LXRα protein were incubated in the absence (ctrl) or presence of increasing concentrations of GGPP and FPP. Binding to an upstream 10,000 bp non-specific sequence was employed as an internal negative control. The bars represent the mean ± s.e.m. of 3 experiments (** $P < 0.01$, *** $P < 0.001$; ANOVA).

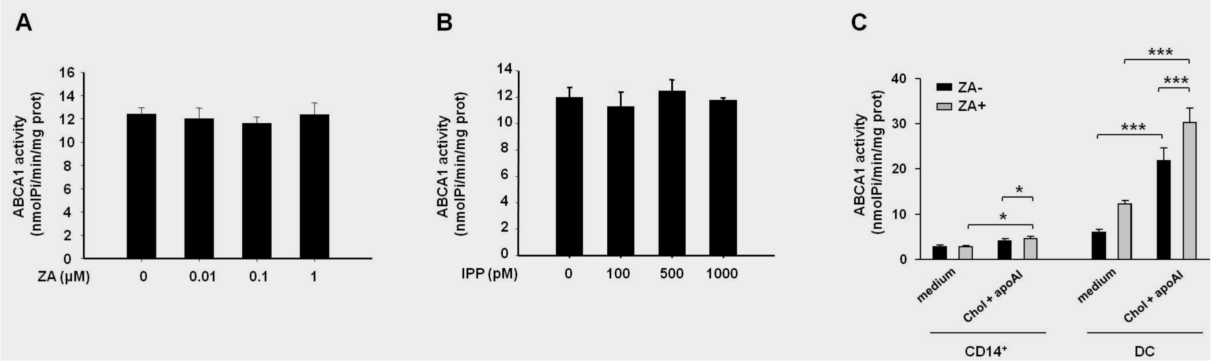

Supplementary Figure 6. Effect of ZA and IPP on ABCA1 activity.

Effect of ZA and IPP on ABCA1 activity; **A-B)** ABCA1 was immunoprecipitated from whole cell lysates of untreated DC. ATPase activity was spectrophotometrically measured on 100 µg of immunoprecipitated proteins that contain an equal amount of ABCA1, in the absence or in the presence of increasing ZA (panel **A**) or IPP (panel **B**) concentrations in the reaction mix. Neither ZA nor IPP modified ABCA1 ATPase activity. The bars represent the mean ± s.e.m. of 4 experiments. **C)** One mg of plasma membrane proteins from $CD14^+$ cells or DC, incubated for 24 h in the absence (ZA-) or in the presence (ZA+) of 1 µM ZA, were subjected to immunoprecipitation with an anti-ABCA1 antibody. The ATPase assay was spectrophotometrically measured on 100 µl immunoprecipitated extracts. DC showed higher ATPase activity than $CD14^+$ cells, which was only increased in ZA-treated DC. For a positive control of maximal ABCA1 activity, 100 µM cholesterol/β-methyl-cyclodextrin (Chol) complexes and 1 µg/ml of human recombinant apoA-I (apoA-I) were added to the ATPase reaction mix. The bars represent the mean ± s.e.m. of 4 experiments (* $P < 0.05$, *** $P < 0.001$; ANOVA).

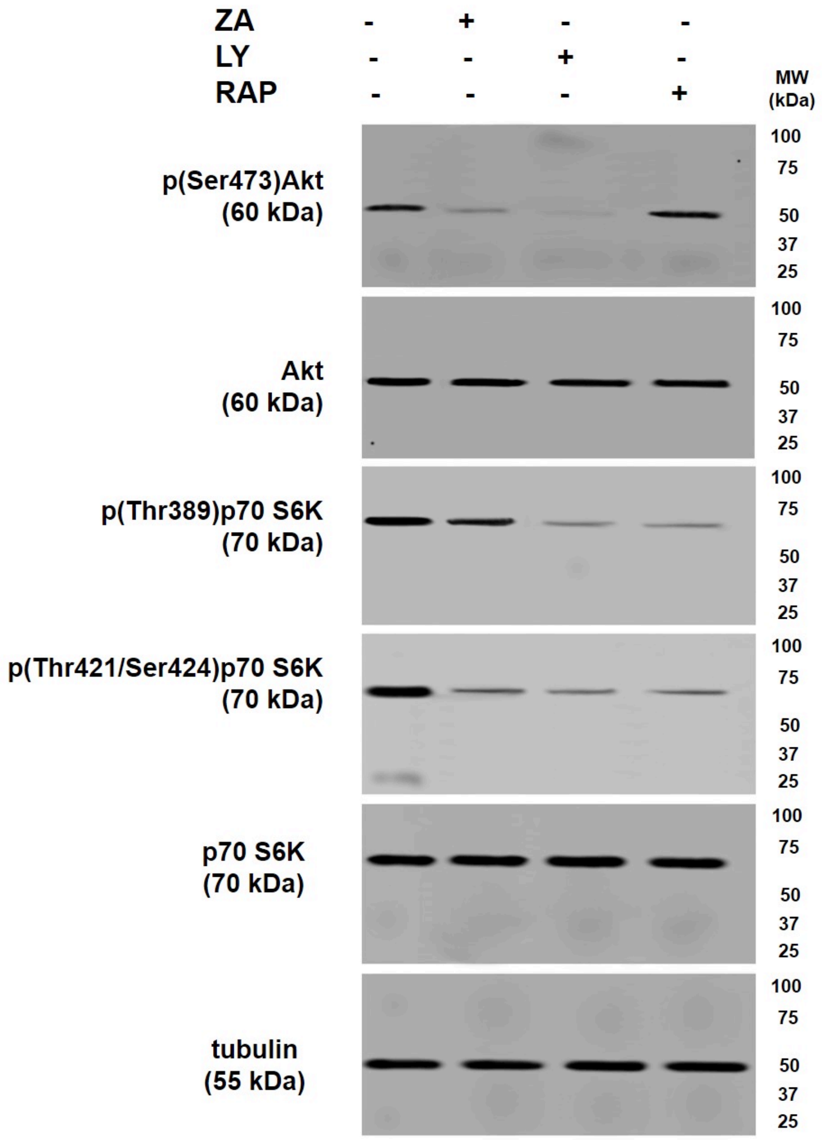

Supplementary Figure 7. Modulation of PI3K/Akt/mTOR signalling pathway in DC.
Western blot analysis of phospho(Ser473)Akt, Akt, phospho(Thr389)p70 S6K, phospho(Thr421/Ser424)p70 S6K, p70 S6K in cell lysates from DC incubated for 24 h in the absence (-) or presence (+) of 1 μM ZA, 200 μM LY294002 (LY) as PI3K inhibitor, and 20 nM rapamycin (RAP) as mTOR inhibitor. The results indicate effective PI3K/Akt/mTOR signalling inhibition by these agents. β-tubulin served as a control for equal protein loading per lane. The blots are representative of one out of two experiments.

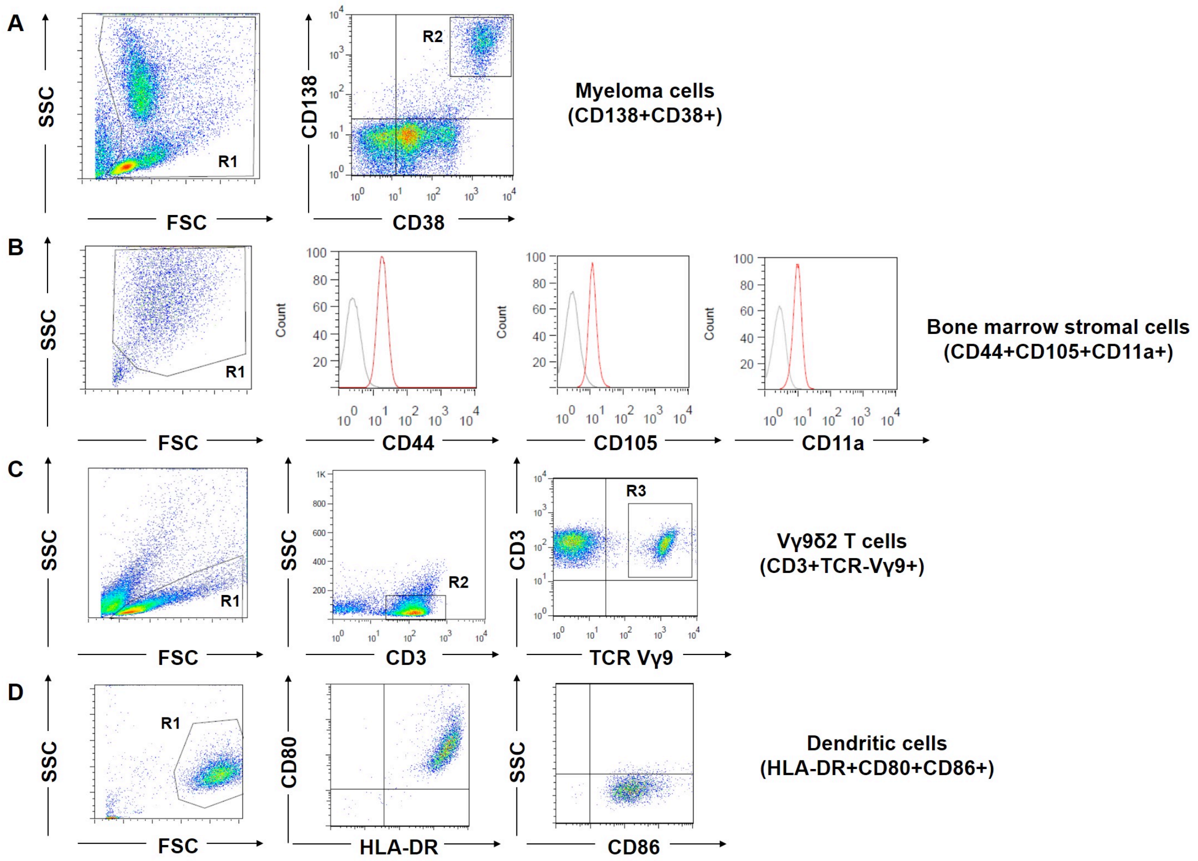

Supplementary Figure 8. Flow cytometry gating strategies.
A) Myeloma cells: the first gate (R1) is drawn to include all viable cells based on FSC vs SSC parameters. Next, cells in R1 are classified as myeloma cells according to double expression of CD38 and CD138 (R2: CD138+CD38+ cells). B) Bone marrow stromal cells: the first gate (R1) is drawn to include all viable cells based on FSC vs SSC parameters. Next, cells in R1 are classified as bone marrow stromal cells according to their expression of CD44, CD105 and CD11a. C) Vγ9Vδ2 T cells: the first gate (R1) is drawn to include all viable cells based on FSC vs SSC parameters. Next, cells in R1 are analyzed for the expression of CD3 to identify the T lymphocyte population (R2: CD3). Finally, Vγ9Vδ2 T cells are gated according to double expression of TCR-Vγ9 and CD3 (R3: TCR-V γ9+CD3+ cells). D) Dendritic cells: the first gate (R1) is drawn to include all viable cells based on FSC vs SSC parameters. Next, cells in R1 are analyzed for the expression of HLA-DR, CD86 and CD80 and classified as dendritic cells according to double expression of HLA-DR and CD80, and the expression of CD86.

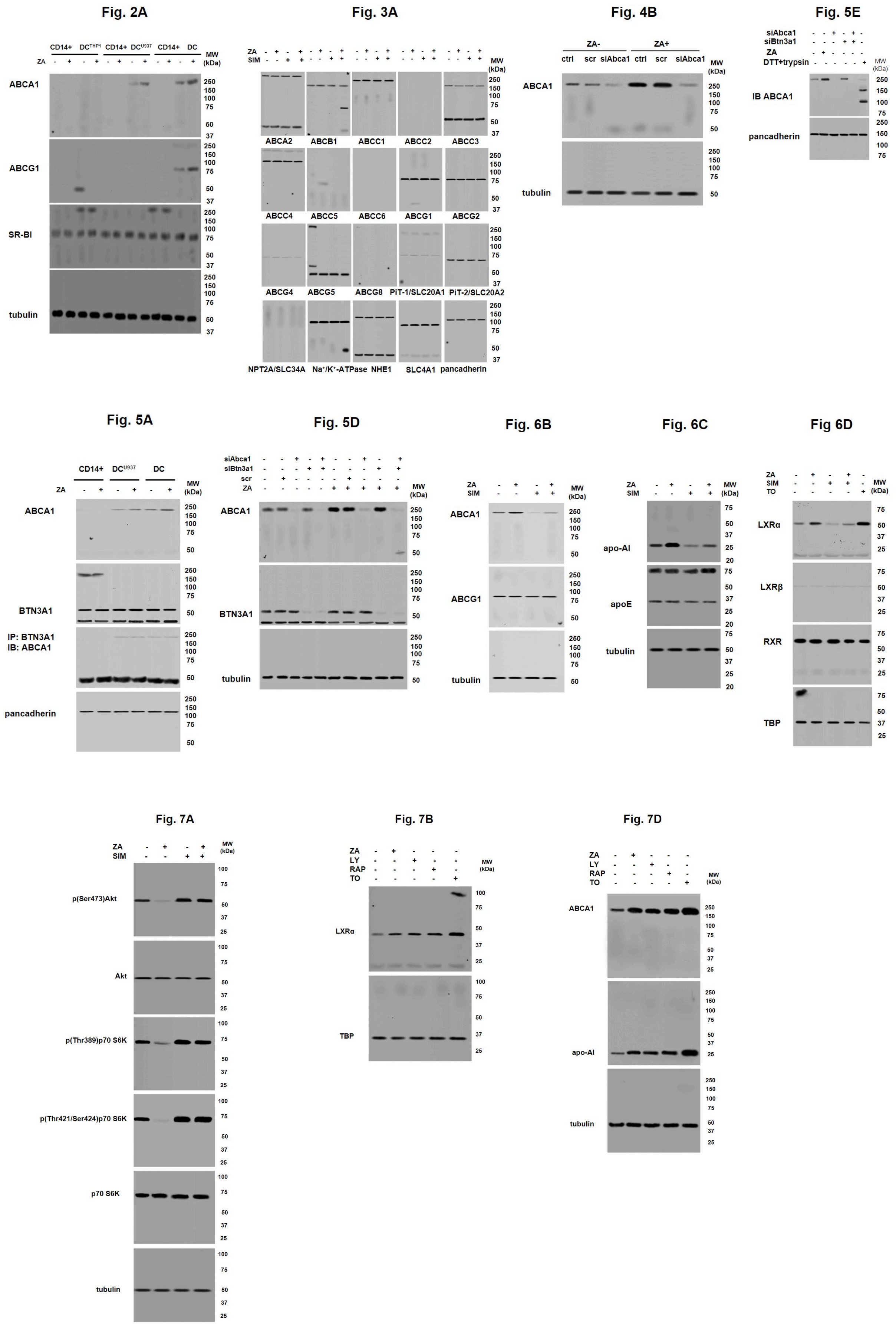

Supplementary Figure 9. Original immunoblotting of main figures.

Fig. S2B

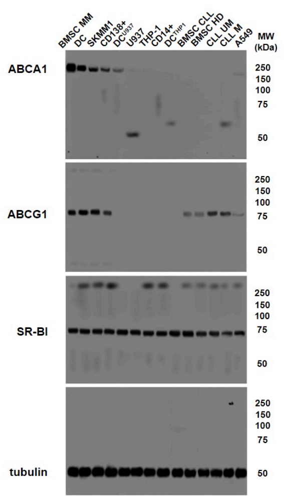

Fig. S4A

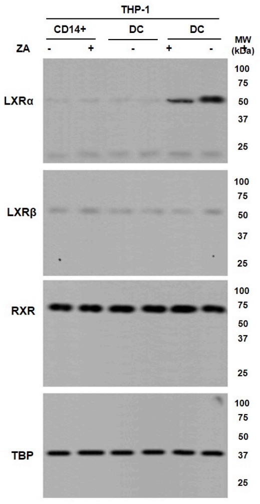

Fig. S3

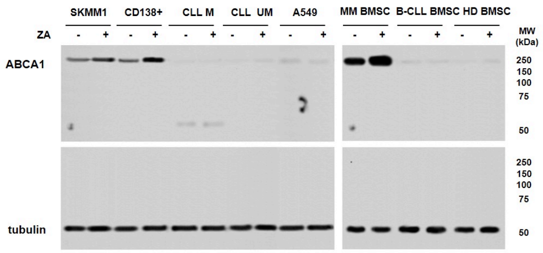

Supplementary Figure 10. Original immunoblotting of supplementary figures.

Band	Result	Accession number	Name	MW	pI	Matched peptides/25	Coverage %	Mascot score
C1	APOE_HUMAN	P02649	Apolipoprotein E	36132	5.65	8	30	67
C2	APOE_HUMAN							51
C3	APOE_HUMAN	P02649	Apolipoprotein E	36132	5.65	12	44	120
C4	APOE_HUMAN	P02649	Apolipoprotein E	36132	5.65	10	38	97
C5	APOA1_HUMAN	P02647	Apolipoprotein A-I	30759	5.56	15	52	192
C6	APOA1_HUMAN	P02647	Apolipoprotein A-I	30759	5.56	17	55	240
C7	APOA1_HUMAN	P02647	Apolipoprotein A-I	30759	5.56	9	34	112
C8	APOA1_HUMAN	P02647	Apolipoprotein A-I	30759	5.56	13	38	150
C9	APOA1_HUMAN	P02647	Apolipoprotein A-I	30759	5.56	8	29	72
C10	APOA1_HUMAN	P02647	Apolipoprotein A-I	30759	5.56	12	47	166
C11	APOA1_HUMAN	P02647	Apolipoprotein A-I	30759	5.56	14	47	194
C12	APOA1_HUMAN	P02647	Apolipoprotein A-I	30759	5.56	14	47	184
C13	APOA1_HUMAN	P02647	Apolipoprotein A-I	30759	5.56	11	36	137
C14	APOA1_HUMAN	P02647	Apolipoprotein A-I	30759	5.56	13	45	170

Supplementary Table 1. ApoA-I and apoE identification in dendritic cell supernatants. Bands were excised from the gel shown in Figure 2C. The excised bands are shown in Supplementary Fig. 1. Slice numbers, accession number for SwissProt database, name, molecular weight (MW), isoelectric point (pI), the number of matched peptides on the number of total peptides searched, sequence coverage and Mascot score are indicated.
